# The effect of attention and working memory on the estimation of elapsed time

**DOI:** 10.1038/s41598-018-25119-y

**Published:** 2018-04-27

**Authors:** Ignacio Polti, Benoît Martin, Virginie van Wassenhove

**Affiliations:** 1 CEA, DRF/Joliot, NeuroSpin; INSERM, U992, Cognitive Neuroimaging Unit, Université Paris-Sud; Université Paris-Saclay, Gif/Yvette, France; 2 Kavli Institute for Systems Neuroscience, Centre for Neural Computation, The Egil and Pauline Braathen and Fred Kavli Centre for Cortical Microcircuits, NTNU, Norwegian University of Science and Technology, Trondheim, Norway

**Keywords:** Attention, Human behaviour

## Abstract

Psychological models of time perception involve attention and memory: while attention typically regulates the flow of events, memory maintains timed events or intervals. The precise, and possibly distinct, roles of attention and memory in time perception remain debated. In this behavioral study, we tested 48 participants in a prospective duration estimation task while they fully attended to time or performed a working memory (WM) task. We report that paying attention to time lengthened perceived duration in the range of seconds to minutes, whereas diverting attention away from time shortened perceived duration. The overestimation due to attending to time did not scale with durations. To the contrary, increasing WM load systematically decreased subjective duration and this effect scaled with durations. Herein, we discuss the dissociation between attention and WM in timing and scalar variability from the perspective of Bayesian models of time estimations.

## Introduction

It is well established that paying attention to a task significantly impairs performance in a concurrent task (e.g. inattentional blindness^1^, a phenomenon generically called dual-task interference^2^). Dual-task interferences are also observed during time estimation, so that being engaged in a concurrent non-temporal task typically shortens the estimated time that has elapsed^3,4^. These observations fit well with the notion that tracking information and tracking time compete for the brain’s limited attentional resources^5^ and, hence, that attention plays a critical role in time estimation^6–9^.

Psychological models of time perception typically include a pacemaker and an accumulator as the central clock, and implicate both working memory and long-term memory^8,10–12^. According to the attentional gate model^13^, attention regulates the transfer of the total amount of pulses counted by the pacemaker to working memory (WM). When attention is diverted away from time, the transfer time from the pacemaker to the accumulator is shortened, ultimately yielding a smaller pulse count in the accumulator (thus in WM) than the one in reference (or long-term memory): this is considered to ultimately yield a shortening of perceived duration. Accordingly, underestimations of duration have been related to the level of difficulty in concurrent non-temporal tasks^14–16^ and to the proportion of attention allocated to non-temporal features of a stimulus^17,18^. In addition to attentional effects, WM resources and tasks involving the central executive typically affect time estimation^19^. For instance, the set size held in WM has been shown to affect the reproduction of duration^20,21^ suggesting a possible implication of WM load on time estimation. Information-theoretic approaches posit that the accumulation of pulses in the accumulator linearly transfers the pulse count to WM and/or to the reference memory for comparison^10,12,22,23^. Durations transferred to WM are thus a final count between the onset and the offset of an interval to be timed^20^. Subjective duration can be seen as the amount of time required to transfer the clock read-out into the reference memory with the pulse accumulation seen as an up-counter, and memory transfer seen as a down-counter^24^. Consistent with this, duration estimation has been reported to increase with the number of stimuli and to decrease with a an increase of performance in a concurrent non-temporal task, with both effects scaling with durations^25^.

Surprisingly however, very few human studies have directly tackled the issue of memory resources despite contrasting interpretations on the functional contribution of WM to timing. WM has been seen as a transfer time constant^24^ but also suggested to scale with timing^26^, leaving open the issue of whether WM simply maintain timing information or functionally intervene in timing *per se*. In the latter, it has been argued that WM may actively contribute to the representation of duration^26^ via a top-down influence of time estimation relying on Bayesian estimation^27^. The Bayesian framework for interval timing offers a different level of description for time estimation in which the estimation of magnitudes is characterized by classic effects (e.g. central tendency, range effects, or scalar variability) which can be explored and experimentally manipulated. These properties will be reviewed and discussed in the discussion section, and will serve as a basis for the interpretation of the empirical effects reported here.

Empirical evidence so far has focused on the effect of WM on the reproduction of time estimation requiring the memory of a previously learned time interval to be reproduced: Fortin and Couture^28^ reported that time reproductions positively correlated with memory set size and a recent study reported that inter-individual WM capacity correlated with an individual’s time reproduction ability^29^. Interestingly, temporal order more than spatial content was shown to affect time reproduction in a study testing whether the underestimations of time was related to the difficulty of a concurrent non-temporal task or to the proportion of attention allocated to non-temporal features of the stimuli^4^. This result suggested that the temporal nature of the information held in memory mattered for interference effects, in agreement with a recent study showing that WM could lengthen subjective durations of content-matching sensory stimuli^30^. In an alternative viewpoint, time intervals could be maintained in WM as would any other chunked informational content, and the effect of WM would be related to the precision with which any information is held in the system. This was suggested by a recent study reporting WM interferences with the estimation of acoustic durations so that the larger the load in WM, the less precise the memory representation for durations^31^.

In light of recent discussions questioning the specific role of memory in timing models^26,27,32^, we thus investigated whether WM could parametrically affect duration estimation, and to which extent the effect of WM and attention could be dissociable. In light of the recent hypotheses regarding the implication of Bayesian computations in the estimation of duration, we were further interested in considering the effects of attention and WM load in the context of a Bayesian model for time perception. To address these, we asked participants to estimate supra-second time intervals of 30 s, 60 s or 90 s. Two main experimental conditions were used in which participants either estimated durations while fully paying attention to time (single-task condition) or while concurrently performing an n-back WM task (dual-task condition).

**Figure 1:**
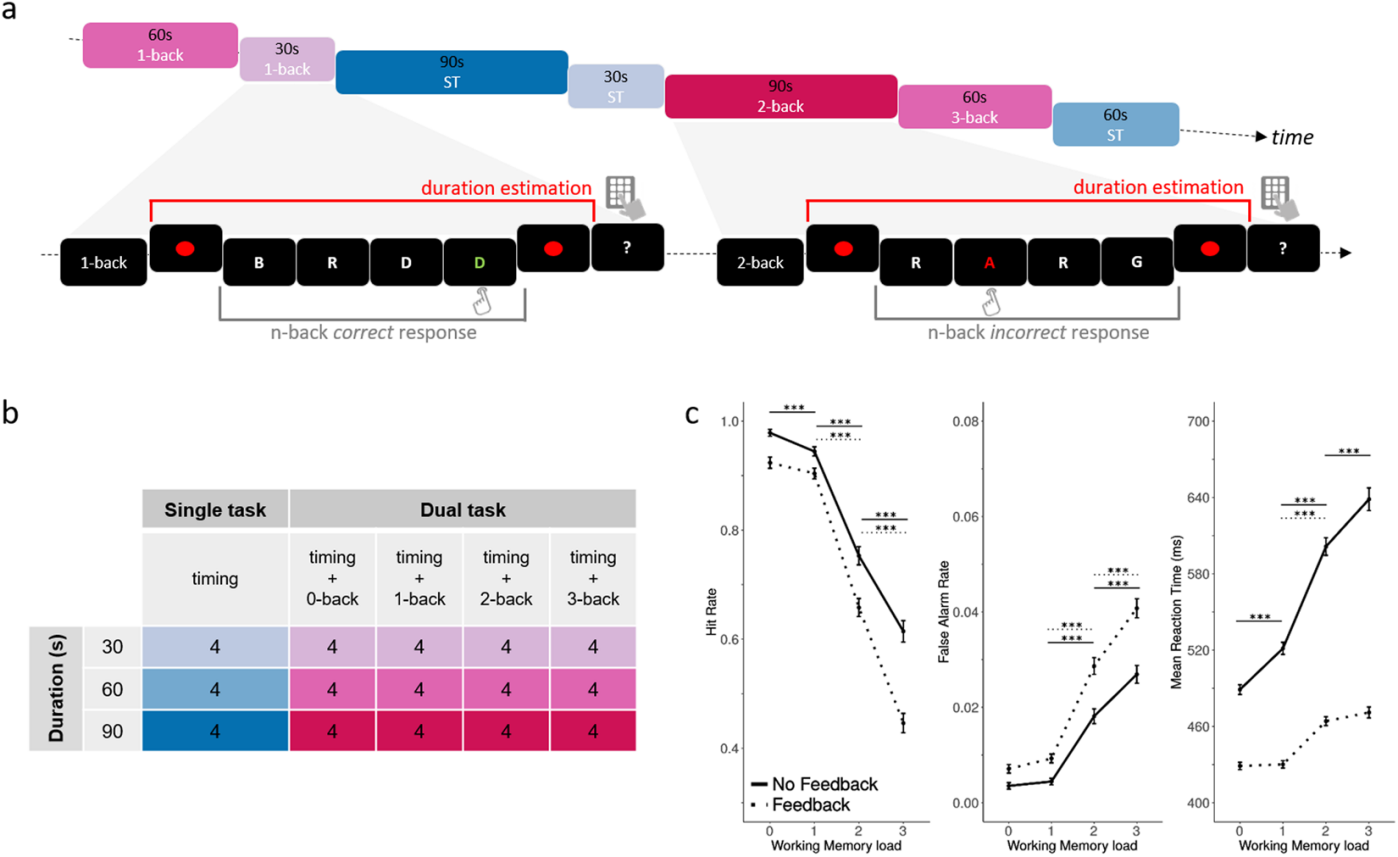
Experimental design and performance on the n-back working memory (WM) task. Panel (a) The experiment consisted in participants performing a prospective duration estimation of 30 s, 60 s, or 90 s. Each target interval was marked by the presentation of a red dot on the screen (thereby delineating a duration trial) and participants provided their duration estimates in minutes and seconds using a numeric pad. Additionally, participants underwent two main experimental conditions: in the single-task (blue shades), they fully paid attention to the elapsed time, whereas in the dual-task condition (red shades) they simultaneously performed a 0-, 1-, 2- or 3-back WM task (n-back). Two groups of participants were tested on all conditions: one group of participants (n = 24) received Feedback on their WM performance (green = correct; red = incorrect) whereas the other group (n = 24) received No Feedback. For further illustration, please see the video example of a dual task trial (30 s duration, 3-back WM) for the Feedback group provided in Supplemental Materials. Panel (b) Both groups of participants performed four duration trials for each possible combination of duration and experimental condition (single-task or n-back conditions in dual-task). Panel (c) The hit rates in the n-back task significantly decreased in both groups as a function of WM load (left graph). Performance was lower in the Feedback group (dotted lines) as compared to the No Feedback (filled lines) group, with a lower hit rate (left graph) and a higher false alarm rate (middle graph) in the Feedback group as compared to the No Feedback group. Right panel: RTs were also slower in the No Feedback as compared to the Feedback group. Error bars indicate s.e.m., ***p < 0.001 (Supp. Tables 1, 2 and 3).

To parametrically assess the effect of WM load on duration estimation in the dual-task condition, we used an n-back WM task in which the n varied from 0 (attentional control in which participants responded to a target stimulus, *i.e*. a simple visual detection task), or 1, 2 or 3 (varying WM load). Here, the hypothesis was that if WM affects the likelihood estimates of duration, the greater the WM load, the shorter the estimated duration. A majority of studies addressing the issue of memory interference with temporal estimation have used temporal reproduction tasks. Here, participants estimated a supra-second duration using explicit numerals, as would naturally be done in real-life, but using a response pad instead of verbalizing durations (Fig. 1). Numerical responses are comparable to the requirements of verbal estimation tasks in that they require the representation of time units^33^ *i.e*. a likely symbolic recoding of temporal estimates. Verbal estimation and time reproduction have been shown to yield comparable results in timing tasks^34^. Additionally, two groups of participants were tested: one group received *Feedback* on their WM performance, the other received *No Feedback*. Feedback on time estimation was never provided. The two groups were tested to insure that the presence of feedback - provided by means of a colour change following the response to the n-back WM task - did not confound the possible parametric changes of duration estimations with WM load. For instance, the higher the WM load, the more errors participants may make and the more colour changes they may experience. Hence, the *No Feedback* group acted as a control for the effect of WM load observed in the *Feedback* group. To build intuition on the paradigmatic approach, a video example of a dual task trial (30 s duration, 3-back WM) for the *Feedback* group is provided in Supplemental Materials.

Overall, we report that both attention and WM load affected duration estimation: paying attention to time lengthened subjective duration, and diverting attention away from duration shortened subjective duration; the attentional effect did not scale with duration. In a Bayesian framework, we propose that attention bias likelihood estimations. To the contrary, WM load parametrically shortened duration estimation and this effect scaled with duration. In a Bayesian framework, we suggest that WM load may contribute to the precision with which duration estimates are maintained.

## Results

### Performance in the n-back WM task with and without feedback

To insure that participants properly performed the WM task during the dual-task conditions, we assessed participants’ performance in the n-back task (Fig. 1c, left panel). As predicted, participants’ hit rate (HR) decreased parametrically as a function of increasing WM load irrespective of feedback (Fig. 1c, left panel; Supp. Table 1: “HRmodel_1”: X^2^ = 801.61, p < 0.001). Supp. Table 2 reports the main effects of WM load on HR separately for the *No Feedback* (Z_0-back vs 1-back_ = 5.914, p < 0.001; Z_1-back vs 2-back_ = 11.187, p < 0.001; Z_2-back vs 3-back_ = 5.823, p < 0.001) and for the *Feedback* group (Z_0-back vs 1-back_ = 0.864, p = 0.8; Z_1-back vs 2-back_ = 13.460, p < 0.001; Z_2-back vs 3-back_ = 9.021, p < 0.001). A significant main effect of Group was found on HR (Supp. Table 1: “HRmodel_2”: X^2^ = 90.765, p < 0.001) showing that providing feedback to participants surprisingly decreased their overall performance in the WM task (Supp. Table 2 for beta regression contrasts). A significant interaction between WM load and Group was also observed (Supp. Table 1: “HRmodel_3”: X^2^ = 18.06, p < 0.001) suggesting that the difference in HR between the two groups increased with WM load (Supp. Table 2, Interaction effect WM load * Group).

Increasing WM load significantly increased the false alarm rate (FA; Fig. 1c, middle panel; Supp. Table 1, “FAmodel_1”: X^2^ = 357, p < 0.001) in the *Feedback* (Supp. Table 2: Z_0-back vs 1-back_ = −1.406, p = 0.5; Z_1-back vs 2-back_ = −10.092, p < 0.001; Z_2-back vs 3-back_ = −4.806, p < 0.001) and in the *No Feedback* group (Z_0-back vs 1-back_ = 0.121, p < 0.9; Z_1-back vs 2-back_ = −8.655, p < 0.001; Z_2-back vs 3-back_ = −4.013, p < 0.01). A main effect of Group was found for FA (Fig. 1c, middle panel; Supp. Table 1, “FAmodel_2”: X^2^ = 53.568, p < 0.001) so that FA were higher when feedback was provided than when it was not (Supp. Table 2: *Main effect of Group*: Z_0-back_ = 2.302, p < 0.05; Z_1-back_ = 3.930, p < 0.01; Z_2-back_ = 4.588, p < 0.001; Z_3-back_ = 5.083, p < 0.001). A significant interaction between WM load and Group was also found (Supp. Table 1, “FAmodel_3”: X^2^ = 9.6927, p < 0.05) so that increasing WM load increased the difference in FA between the two groups (Supp. Table 2, Interaction effect WM load * Group).

Finally, reaction times (RTs; Fig. 1c, right panel) significantly increased with increasing WM load (Supp. Table 3, “RTmodel_1”: X^2^ = 577.25, p < 0.001) in both groups: with feedback (Supp. Table 2: t_0-back vs 1-back_ = −1.609, p = 0.9; t_1-back vs 2-back_ = −6.101, p < 0.001; t_2-back vs 3-back_ = −1.443, p = 0.4) and without feedback (Supp. Table 2: t_0-back vs 1-back_ = −5.668, p < 0.001; t_1-back vs 2-back_ = −13.740, p < 0.001; t_2-back vs 3-back_ = −6.722, p < 0.001). Participants who did not receive feedback were faster at detecting the targets (Supp. Table 3, “RTmodel_2”: X^2^ = 45.319, p < 0.001; Supp. Table 2: *Main effect of Group*). A significant interaction between WM load and Group was also found (Supp. Table 3, “RTmodel_3”: X^2^ = 185.09, p < 0.001), in which the difference in RTs between the two groups scaled with WM load (Supp. Table 3, Interaction effect WM load * Group).

Overall, these results suggest that participants readily engaged in the n-back WM task so that the required experimental manipulation of WM load was fulfilled. The differences between the effect of feedback suggests that the engagement of participants may have been differentially impacted by the ongoing duration estimation they knew had to be realized. In other words, the detrimental effect of feedback on WM performance may be related to the dual engagement of participants in timing. However, this observation is outside the scope of the study and it may be interesting in subsequent work to investigate the possible bidirectionality of interference effects between timing and WM tasks. We now focus solely on the interferences of WM and attention on duration estimation.

### Effect of attention on prospective duration estimation assessed with single *vs*. 0-back dual-task conditions

**Figure 2:**
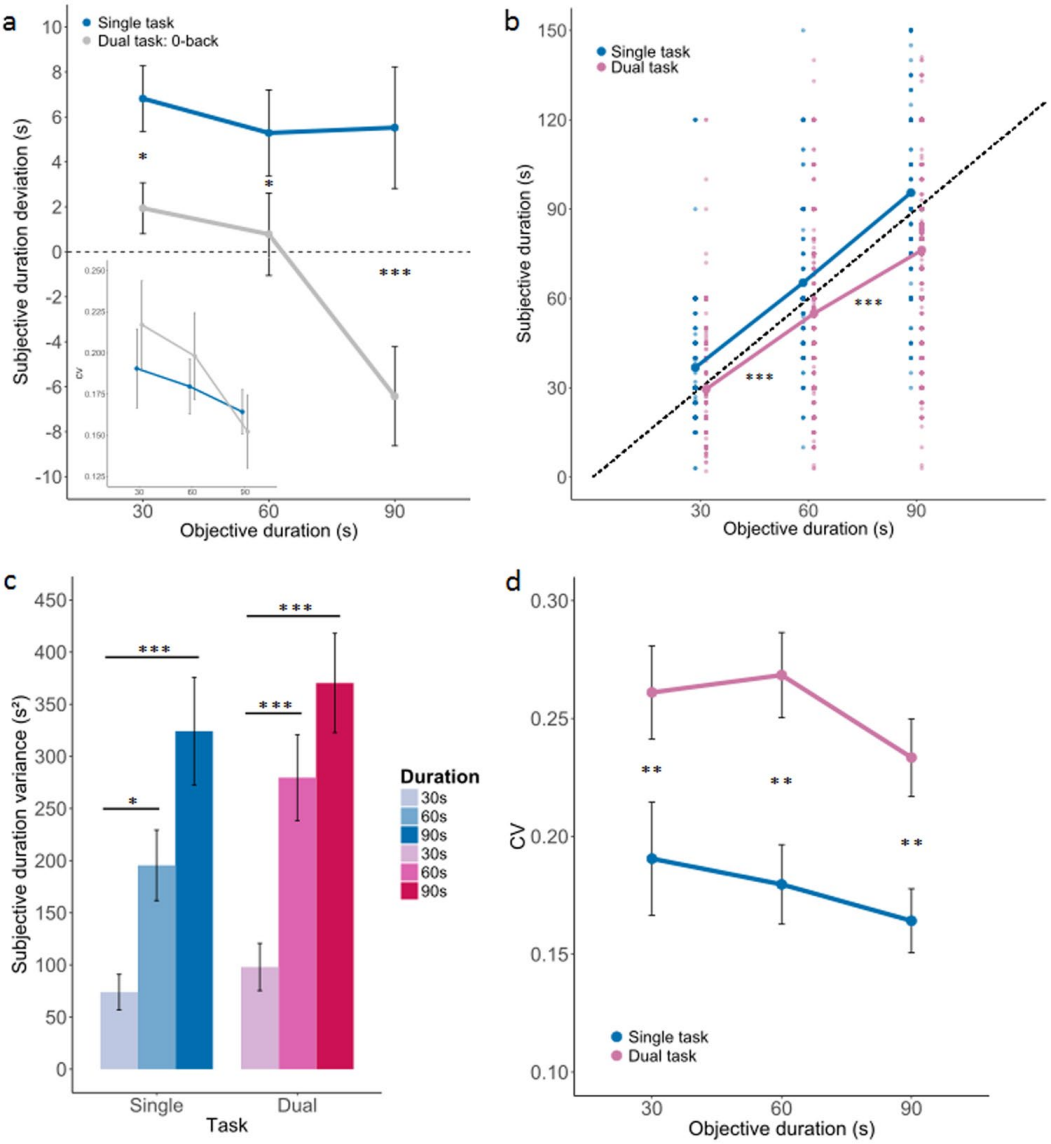
Duration estimation in single-task and dual-task conditions. Panel (a) The subjective duration during single-task (blue) was mostly overestimated as compared to veridical duration (the zero line). In the 0-back dual-task condition (grey), duration estimates were shorter than during the single-task condition. The difference of duration estimation between single- and dual-task conditions was comparable (~5 seconds) whether the veridical duration was 30 s, 60 s or 90 s (Supp. Fig. 1 and Supp. Table 4). The inset plot provides the Coefficients of Variation for each duration in single (blue) and 0-back dual-task conditions (grey). Panel (b) The scatter plot reports the subjective duration estimates in single-task (blue) and dual-task (pink) as a function of the three target durations (30 s, 60 s and 90 s). One dot is an individual’s duration estimate (a darker dot thus signifies that many individuals share a similar duration estimate). The dashed line represents the veridical line for which subjective duration would be identical to objective duration. In single-task, the allocation of attention to timing yielded a significant overestimation of duration across all three durations. No significant effect of duration was found for this overestimation so that the magnitude of overestimation was constant across durations. In the dual-task condition, a significant underestimation of duration was found across all three durations. The underestimation of duration scaled with duration so that the longer durations were significantly underestimated as compared to the shorter durations, irrespective of working memory load. Panel (c) The variance of duration estimates predictably scaled with duration in agreement with scalar variability. This was observed in single (blue) and in dual (red) task conditions. The single-task *vs*. dual-task conditions did not significantly affect the variance for each duration estimates. Panel (d) The coefficient of variations (CV) did not significantly differ between durations irrespective of single-task and dual-task conditions. However, the CV significantly differed across single-task and dual-task conditions, with the CV in the dual-task being larger than in the single-task. These results, combined with results in panel (b), suggest that the WM task may have an impact on the likelihood estimates of duration. Error bars indicate s.e.m., *p < 0.05, **p < 0.01, ***p < 0.001.

In the single-task condition, all durations were overestimated as compared to objective durations (Fig. 2a, blue) whereas durations went from near veridical/overestimated to underestimated in the 0-back dual-task condition (Fig. 2a, grey). As the 0-back condition consisted in pressing the button for each target letter appearing on the screen, this condition provided an attentional control allowing us to compare paying full attention to time (single-task) with paying attention to a visual detection task with minimal cognitive load as compared to the n-back conditions subsequently tested. The time estimates were transformed to express the amount of deviation from the target duration (as Δt = subjective duration - veridical duration). Using this approach, positive values could be interpreted as over-estimations and negative values conservatively as under-estimations (both being in comparison to veridical duration or unbiased ideal observer).

An lme was performed to test whether Duration (30 s, 60 s, 90 s) and Task (single-task, 0-back dual-task) predicted time estimation biases. A main effect of Task was found (Fig. 2a; Supp. Table 3, “STvsDT0-back_model_1”: X^2^ = 29.419, p < 0.001), suggesting that during the single-task condition, durations were significantly overestimated as compared to the 0-back dual-task condition (Supp. Table 4, Main effect of Task). During the single-task condition, 30 s intervals were overestimated on average ~7 s (SE = 2.65 s), 60 s intervals were overestimated ~6.5 s (SE = 2.66 s) and 90 s intervals were overestimated ~7 s (SE = 2.69 s). Although we did not find a main effect of Duration (Supp. Table 3, “STvs.DT0-back_model_2”: X^2^ = 5.266, p = 0.07; Supp. Table 4), we found a significant crossover interaction (Szklo & Nieto, 2014) between Task and Duration (Table 3, “STvs.DT0-back_model_3”: X^2^ = 6.322, p < 0.05; Supp. Table 4). Data are reported in Supp. Figure 1a and statistical contrasts in Supp. Table 4. Underestimation increased as a function of duration in the 0-back dual-task condition (30 s *vs*. 60 s: t = 0.128, p = 0.99; 30 s *vs*. 90 s: t = 3.077, p < 0.01; 60 s *vs*. 90 s: t = 2.894, p < 0.05), but no significant differences in overestimation were found in the single-task condition (30 s *vs*. 60 s: t = 0.269, p = 0.96; 30 s *vs*. 90 s: t = 0.046, p = 0.99; 60 s *vs*. 90 s: t = −0.219, p = 0.97). In other words, paying attention to duration lengthened subjective duration, but this lengthening did not scale with target duration. In fact, this effect either increased or decreased with longer durations according to the experimental groups (Supp. Fig. 1b). Given that the single-task condition was identical in both groups (in this condition, there was no feedback provided to the *No Feedback* or to the *Feedback* groups), the variance observed here may be accounted for by random inter-individual variability.

The pattern of results thus suggests that the internal representation of duration estimation would be somewhere between the blue and the grey data, with attention effectively acting as an up and down switch of this internal estimation; interestingly, the effect of paying attention to time did not robustly increase with the length of duration. To directly test this, we computed the coefficient of variations (CVs) separately for each duration in the single-task and in the 0-back dual-task condition (Fig. 2a, inset). No significant differences were found as a function of Duration (Supp. Table 3, “CV_STvs.DT0-back_model_1”: X^2^ = 4.4926, p = 0.11) or as a function of Task (Supp. Table 3, “CV_STvs.DT0-back_model_1b”: X^2^ = 0.6481, p = 0.42), thus suggesting that paying attention – or not paying attention (to time would not affect the scalar property or the precision of duration estimation.

### Effect of single- and dual-tasks on time estimation

In the dual-task conditions (including all n-back conditions), duration was overall significantly underestimated (Fig. 2b, pink; Supp. Table 3, “Dev_SvsDtask_model_2”: X^2^ = 120.53, p < 0.001; Supp. Table 5, *Main effect of Duration*). Unlike the attentional effect described above, the underestimation significantly scaled with the target durations so that 30 s intervals were estimated on average to be ~29 s (SE = 2.17 s), 60 s intervals were estimated as ~55 s (SE = 2.18 s) and 90 s intervals were estimated as ~77 s (SE = 2.19 s). Comparing the amount of underestimation across durations yielded systematic significant differences (30 s *vs*. 60 s: t = 4.239, p < 0.001; 30 s *vs*. 90 s: t = 12.192, p < 0.001; 60 s *vs*. 90 s: t = 7.936, p < 0.001). Consistent with the above observations, significant differences between the single- and the dual-task conditions were observed (Supp. Table 3, “Dev_SvsDtask_model_1”: X^2^ = 155.28, p < 0.001; Supp. Table 5, *Main effect of Task*) independently for each duration (t_30s_ = 4.678, p < 0.001; t_60s_ = 6.615, p < 0.001; t_90s_ = 11.548, p > 0.001). As could be expected, a significant interaction between Task and Duration was observed so that differences between single- and dual-task conditions increased with longer durations (Supp. Table 4, “Dev_SvsDtask_model_3”: X^2^ = 27.31, p < 0.001; Supp. Table 5, Interaction Task * Duration). This effect was driven by the scaling effect observed in the dual-task condition as a function of duration. The full data for the regression model are reported in Supp. Fig. 2.

### Effect of single- and dual-task on the variance of time estimation

The scalar property of time estimation is the empirical observation that the variance of subjective durations scale with duration. In Fig. 2c, we report the variance observed during single-task (blues) and dual-task (pinks) as a function of duration intervals: as predicted, we observed a significant increase of the variance with increasing duration. To test whether the scaling induced by WM affected the scalar property of timing during dual-task, we fitted an *lme* model using the variance computed from all subjective duration estimates as dependent variable. As factors, we included Duration (3 levels: 30 s, 60 s, 90 s) and Task (2 levels: single-task and dual-task). As predicted, this analysis revealed a main effect of Duration (Supp. Table 3, “Var_SvsDtask_model_1”: X^2^ = 70.994, p < 0.001; Supp. Table 6, *Main effect of Duration*) so that, consistent with scalar variability, the variance of subjective duration estimates increased for both 60 s and 90 s durations when compared to 30 s durations. This was observed during single-task and during dual-task (ST: t_60s−30s_ = 2.475, p < 0.05; t_90s−30s_ = 5.125, p < 0.001; t_90s−60s_ = 2.652, p < 0.05; DT: t_60s−30s_ = 3.829, p < 0.01; t_90s−30s_ = 5.741, p < 0.001; t_90s−60s_ = 1.912, p = 0.1376).

We then computed the coefficient of variations (CVs) separately for each duration, in the single- and dual-task conditions (Fig. 2d). To test whether the CVs changed as a function of single- or dual-task condition and as a function of duration, we performed an *lme* model including a by-subject random slope for the effect of Task (Supp. Table 3, “CV_SvsDtask_model_1”: X^2^ = 29.348, p < 0.001). We found that the CVs in the single-task condition were significantly smaller than in the dual-task conditions across all durations (Supp. Table 7, Main effect of Task: t_30s(ST–DT)_ = −3.058, p < 0.01; t_60s(ST–DT)_ = −3.826, p < 0.01; t_90s(ST–DT)_ = −2.932, p < 0.01). This pattern suggests that WM may interfere with duration estimation in a manner consistent with the hypothesis that the precision of estimated duration held in WM memory may be modulated^31^. To test this hypothesis, we thus turned to the effect of WM load on prospective duration estimation.

### Prospective duration estimation is parametrically affected by WM load

**Figure 3:**
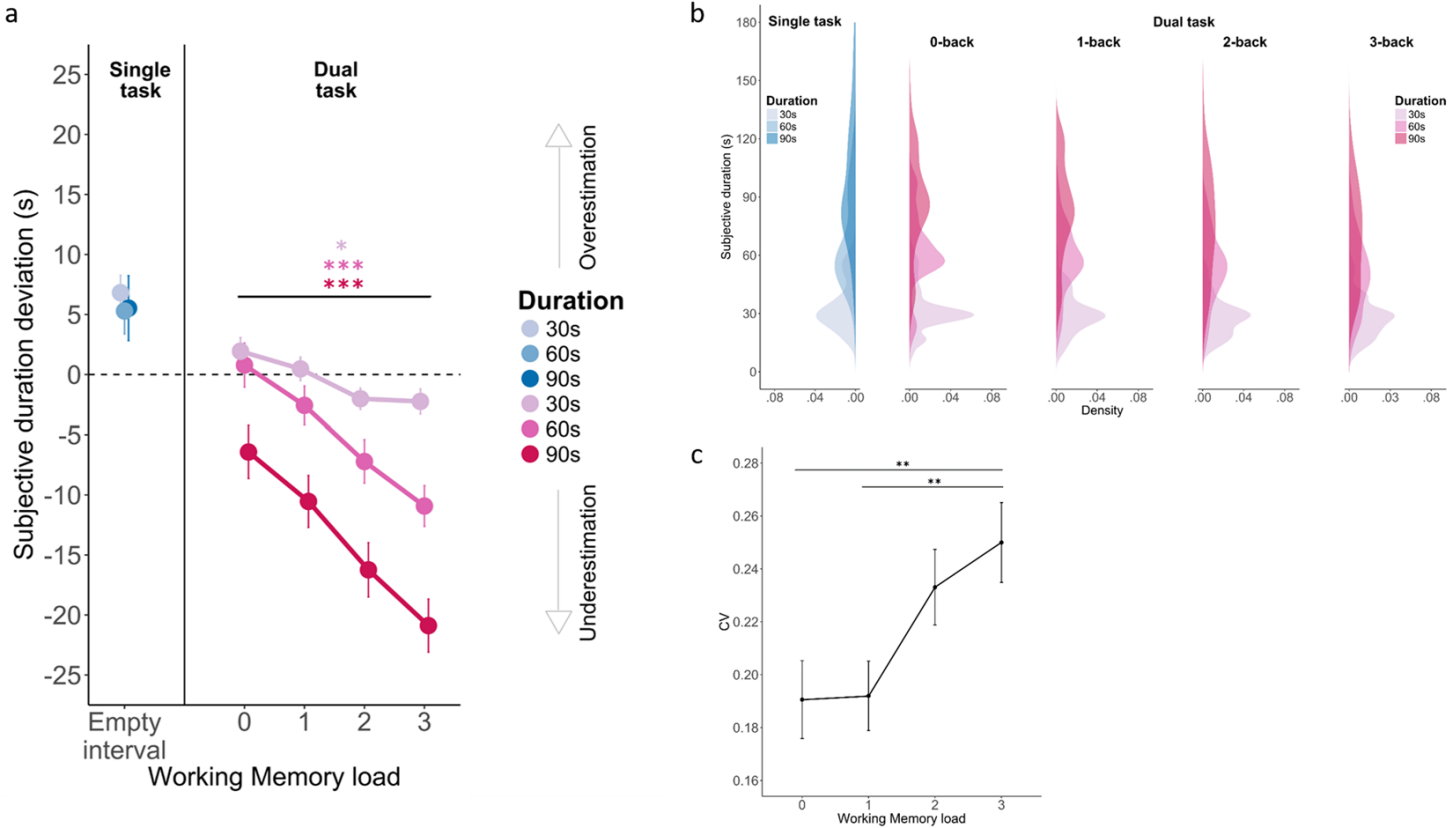
WM load incrementally affects duration estimation. All data illustrated here combined the *Feedback* and *No Feedback* groups who showed comparable effects. Their separate analysis is provided in Supp. Figure 1. Panel (a) Subjective duration estimates were transformed to express the amount of over/under-estimation in the single-task (blue) and in the dual-task (pink) as a function of WM load conditions. Hues are durations with lighter (darker) hues marking shorter (longer) durations. The dashed line represents the ideal observer so that positive values indicate a subjective overestimation of duration and negative values indicate a subjective underestimation of duration. Irrespective of feedback (cf. Supp. Figure 1), durations were overestimated in single-task and underestimated in dual-tasks. Remarkably, the underestimation of duration in dual-task condition systematically increased with WM load. Panel (b) the distribution of duration estimates is plotted as a function of duration (30 s, 60 s, 90 s), single-task (blue, left graph) and dual-task (red) and WM load. In both single and dual-tasks, the response variance significantly increased with duration in agreement with the scalar property. Interestingly the peak and width of the distributions varied as a function of WM load. Panel (c) Increased CV as a function of WM load. Error bars indicate s.e.m., *p < 0.05, **p < 0.01, ***p < 0.001.

Here, we focused our analysis on the effect of WM load (4 levels: 0-back, 1-back, 2-back and 3-back) and Duration (3 levels: 30 s, 60 s, 90 s) on the estimation of duration in dual-task. For this, we fitted an *lme* model using the above-mentioned factors as fixed effects. The underestimation of duration was found to increase with higher WM loads (Supp. Table 3: “WM_model_1”: X^2^ = 124.29, p < 0.001; Supp. Table 8, *Main effect of WM load*). A main effect of duration was found in both the *Feedback* and in the *No Feedback* groups (Supp. Table 3, “WM_model_2”: X^2^ = 181.58, p < 0.001): on average, and irrespective of WM load, longer durations were underestimated more than shorter durations (Supp. Table 8, *Main effect of Duration*). We also found a significant interaction between WM load and Duration (Supp. Table 3, “WM_model_3”: X^2^ = 26.736, p < 0.001; Supp. Table 8, Interaction WM load * Duration). This interaction suggested that the differences in the underestimation of duration across target durations increased with higher WM load. Figure 3a provides a synthetic view of the main effects of attention and WM load combined in both experimental groups. Supp. Figure 3 provides the same data sorted as a function of the *Feedback* and the *No Feedback* group.

### Effect of WM load on duration estimation and coefficients of variance

In Fig. 3b, we illustrate the variance of duration estimation in both experimental groups (n = 48) as a function of WM load in the dual-task conditions (pink). The distribution observed in the single-task (blue) are also provided for comparison. The shift in the peak distribution during the dual-task can readily be seen to vary with the n-back task across all three durations. We thus asked whether the CVs were affected by WM load. Figure 3c reports the CVs as a function of WM load. The CVs were fitted with an *lme* model using WM load as fixed effect. A main effect of WM load was found (Supp. Table 3, “CV_WMload_model_1”: X^2^ = 16.05, p < 0.01): in the dual-task condition, CVs significantly scaled with increasing WM load (Sup. Table 9, Main effect of WM load: t_0-back–3-back_ = −3.300, p < 0.01; t_1-back–3-back_ = −3.203, p < 0.01). This showed that WM-load affected the CVs irrespective of the duration.

## Discussion

**Figure 4:**
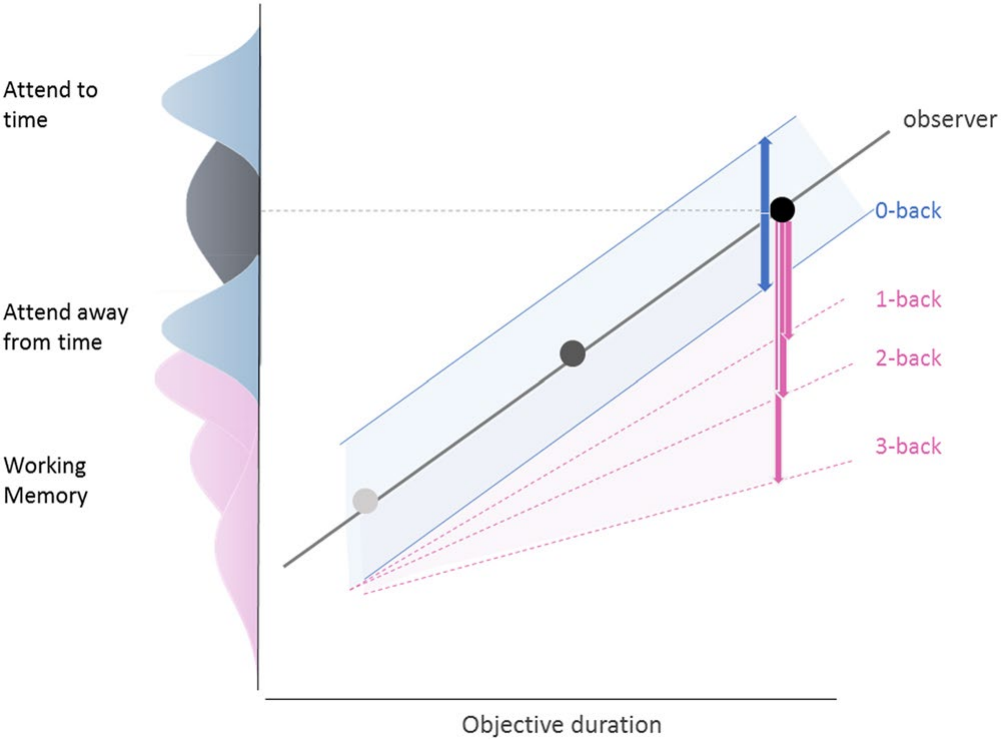
Summary of attention and WM load interferences from the perspective of Bayesian time estimation. First, attention (blue) may systematically shift the likelihood distribution of the original target duration prior (grey): paying attention to time may shift the distribution towards larger estimates resulting in an overestimation of the duration (upper blue, posterior) whereas diverting attention away from time may shift the distribution towards shorter estimates (bottom blue, posterior). Importantly, our results suggests that the precision of under- and over-estimations of duration due to attention is comparable across the full duration range. To the contrary, WM may skew the likelihood distribution as a function of duration so that WM load would affect both the mean and the width of likelihood distributions in addition to scaling with duration. This would result in increasingly wider posterior distributions shifted towards shorter durations (Fig. 3c for empirical data).

Our behavioral study explored the effect of attention and working memory load on prospective duration estimation. Our main findings are that, (i) paying attention to the estimation of duration lengthens subjective duration; (ii) splitting attention to a concurrent WM task shortens perceived duration; (iii) the magnitude of attentional over-estimation was comparable across durations in single-task; (iv) attention did not affect CVs; (v) performing a concurrent WM task shortened perceived duration proportionally to the WM load; (vi) the effect of WM scaled with duration so that shorter durations were less affected by WM than longer durations; (vii) WM load affected timing precision equally across the three target durations so that an increase in WM load also increased the coefficient of variations (CVs). We discuss the implications of these findings in the context of a Bayesian framework for time estimation with the main effects synthesized and compiled in Fig. 4.

Three major features of time perception are the central tendency, the range effect, and the scalar variability^35^. *First*, the central tendency or regression to the mean effect, is the observation that when a range of durations has to be reproduced, the shorter durations tend to be overestimated whereas longer durations tend to be underestimated (also known as Vierordt’s law, *cf*.^36^)^10^. The central tendency effect is now considered a signature of Bayesian computations in the estimation of magnitudes such as duration^35,37–41^. *Second*, given a range of durations, the central tendency is more pronounced for longer than for shorter durations^37^, suggesting that the estimation of duration is shaped by context. For instance, recent findings have demonstrated the existence of carry-over effects in duration estimation, which were accounted for by the combination of perceptual and decisional biases induced by the preceding context^42,43^. Altogether, regression to the mean and range effects have been suggested to emerge from the need to minimize errors in a noisy decision process governed by Bayesian computations^37–40,43,44^.

The estimation of duration or interval timing in this context is realized by taking into account the likelihood estimates of the elapsed time (*i.e*., in our task, the time interval between the two red dots), and the prior for the given duration, that is the knowledge or memory of the to-be-estimated duration shaped by the context. In our study, manipulating attention to time appeared to have an effect comparable to error minimization due to context: overall, shorter durations tended to be overestimated and longer durations tended to be underestimated whether paying full attention to time in the single-task or being in a dual-task condition (Fig. 2a). Additionally, paying attention to time affected duration in a comparable manner across target durations with no change in precision (as assessed by CVs).

One possible explanation, consistent with a general role of attention as gain modulation^45^, would be that the likelihood estimates for a given duration may be shifted towards longer durations when paying attention to time, but towards shorter durations when attention is diverted away from it (Fig. 4). This pattern held, irrespective of target durations: in the single-task condition, paying attention to time contributed to an overestimation of duration with a comparable precision as paying attention away from time, which yielded underestimation. One possible role of attention in a Bayesian framework of time estimation may thus be to bias up or down the likelihood estimations of elapsed time, which would generally be consistent with the traditional role of attention in internal clock models as modulating the on/off switch^6–9^.

One alternative could be that attention affects the decisional criterion so that evidence accumulation may reach a duration criterion at an earlier or at a later latency according to the participant’s attentional orientation (diverted away from, or focused on time, respectively). Considering that attention did not scale with estimated duration in the single-task, and did not affect the CVs in the single-task or in the 0-back dual-task, why the decision criterion would not scale with duration remain puzzling. Altogether, neither the central tendency nor the range effect seemed to be differentially impacted by attention in this task. Follow up studies would thus be very helpful in determining the conditions under which the effect of attention could scale with the range of durations being used and whether attention can be conceived as a bias or gain function of duration likelihood estimations.

The *third* property of magnitude estimation, and interval timing in particular, is the scalar variability consisting in the observation that variance scales with duration, i.e., the noise of the representations scales with its duration^46^. Scalar variability *“[…] means in essence that a source of random variability, not in itself scalar, induces scalar expression of that variability at different target times because the mechanism underlying performance entails a rescaling of the random variable by the target time”*^46^. Said differently, scalar variability is the property of noisy representations onto which the brain computes^47^. In the scalar timing theory^12^, scalar noise characterizes time representations, and more generally magnitude representations^39^. Consistent with this general property of timing, we report scalar variability in both single- and dual-tasks when using a numerical estimation of duration in the supra-second range.

Additionally, the WM load parametrically shortened perceived durations so that the higher the n-back, the more underestimated subjective durations were. This effect scaled with durations so that the WM load affected shorter durations less than it affected longer durations, suggesting that the WM load may not have only skewed the likelihood estimates of elapsed time but also affected their variance. In this study, this effect is quite distinct from the attentional effect. In a Bayesian framework, this would amount to the likelihood of the duration estimates scaling with memory load (Fig. 4). According to timing models, and within a Bayesian framework, the multiplicative factor accounting for scalar variability could intervene at the likelihood estimation stage, or at the transferred posterior, which would be mechanistically equivalent to memory scaling. Previous modeling approaches of time estimation have, by default, assigned scalar variability at the stage of the likelihood estimation^37–40,43,44^ although, as previously discussed, the Bayesian framework makes no assumption regarding the scalar property of timing^35^. In other words, the origin of scalar variability in Bayesian models of time estimation does not constitute a functionally relevant variable in time computation and rather been assigned as an *ad-hoc* property of interval timing. Scalar variability has generally been considered as the noise of remembered representations which either originates from memory itself, or from its read out, but not from the measurement of the magnitude *per se*^47^. In scalar timing theory^48^, the scalar property would originate from a scaling factor which is multiplied to the experienced time interval and whose origin is also tied to memory^35^. Our results thus support the notion that manipulating WM load while timing scales with the estimation of duration. Specifically, the effect of WM on duration estimation quantified here appears consistent with the notion of *precision* in the representation of duration: for instance, scalar property may emerge from the iterative assignment of the scalar factor in the course of the experienced duration, due to the maintenance of duration estimates in WM. This could also occur at a later decisional stage through direct comparison between the stored duration and the WM output. The observed scaling of duration estimation with WM load is in line with the notion that the representation of duration scales with noise, but also with alternative interpretations suggesting that the representation of duration in memory may be subject to deterioration in precision with increased WM load^31^. In a recent study, the source of scalar variability was proposed to put a limit on the precision with which quantities may be represented in the brain^49^, and this proposal appears consistent with the observation that increased information held in WM would interfere with the precision of quantity representation.

The combination of computational approaches and neuroimaging would help make the case on the distinct effects of attention and WM load on time estimation. Although many brain regions engaged during timing overlap with attention and memory networks^50,51^, recent fMRI evidence has also shown some selective engagement of cortical regions during temporal estimation tasks [e.g.^52^]. Functionally, recent hypotheses have also emerged suggesting that oscillatory multiplexing may contribute to the precision of maintained duration estimation^53^. The prediction that behavior alone could not disentangle the possible physiological implementations of the scalar property was previously raised with the hypothesis that the scalar property may result from a computational sampling procedure between memorized *event* timing as opposed to *duration* retrieval^46^. Whether the attentional gain regulation, and the time-information scaling trade-off reported here implicate neural oscillations thus remain to be tested.

## Methods

### Participants

24 subjects (12 males; mean age = 25.1 ± 1.9 years old) were included in the *Feedback* group; 24 new subjects (11 males; mean age = 26 ± 6 years old) were included in the *No Feedback* group. All were right-handed with corrected-to-normal vision, no history of psychological disorders and all were naive as to the purpose of the study. All participants were compensated for their participation. All experimental methods were carried out in accordance with the relevant guidelines and regulations, and the experimental protocol was approved by the Commissariat à l’Energie Atomique et aux Energies Alternatives (CEA, DSV/I^2^BM, NeuroSpin, Gif-sur-Yvette, France). All participants provided written informed consents in accordance with the Ethics Committee on Human Research at the Commissariat à l’Energie Atomique et aux Energies Alternatives (CEA, DSV/I^2^BM, NeuroSpin, Gif-sur-Yvette, France) and the declaration of Helsinki (2008).

### Experimental Design and Procedure

The experimental task was designed in Python using the Pygame library (http://www.pygame.org/). Participants performed a prospective duration verbal estimation task of 30 s, 60 s or 90 s in a single-task condition (Fig. 1a, ST) or in a dual-task condition (Fig. 1a, n-back). In the single-task condition, participants solely performed a duration estimation favoring the full deployment of attention towards timing. In the dual-task condition, participants concurrently performed a duration estimation and an n-back working memory (WM) task. This experimental manipulation invited participants to split attention between timing and WM. In the dual-task, a typical trial consisted of a visual n-back WM task during the entire length of the duration trial.

In the n-back WM task, visual stimuli were white 50-point Arial font capital letters centered on a grey background. A stream of letters was built by connecting several random-generated-letter chunks of 10 stimuli each and a target letter was placed in a pseudo-random position on each of these chunks to ensure a uniform deployment of attention on the n-back task. Each letter was presented for 600 ms, and inter-stimulus intervals were 500 ms long. Four levels of n-back were used: in the 0-back condition, participants were asked to detect the letter ‘C’ in a stream of letters presented successively on the screen by pressing the space-bar (attention control condition). In the 1, 2 and 3-back conditions, participants were asked to press the space-bar when the current stimulus and the stimulus in the n^th^ position before it were identical (the higher the n^th^ position, the higher the memory task demands). Each group of participants performed different versions of the n-back task: participants in the *Feedback* group were provided with response feedback and whenever the participant’s response was a hit, the letter displayed on the screen would turn green, otherwise, it would turn red (error); in the *No Feedback* group, no feedback was provided to participants whatsoever.

Participants were comfortably seated 80 cm away from a Viewsonic CRT monitor (19′′, 60 Hz) in a darkened soundproof cabin. After a first training block on the n-back task, participants were asked to estimate the length of time during which they were engaged in the task, and which was bounded by two red dots at the beginning and at the end of each duration trial. Each dot was displayed on screen for 1 s. Participants were asked to use the number keypad to provide their time estimates by entering 4 digits to validate their response: 2 digits corresponding to the number of minutes, and 2 digits for the number of seconds (for instance, after a 90 s block, an ideal participant would enter 01:30). In the course of the session, three possible trial durations were tested and could be 30 s, 60 s or 90 s long. No feedback was provided regarding subjective time estimates in the *Feedback* or in the *No Feedback* group. A video example of a dual task trial (30 s duration, 3-back WM) for the *Feedback* group is provided in Supplemental Materials.

All possible combinations of n-back WM block and duration were tested four times per participants for a total of 48 trials per experimental condition (Fig. 1b). Trials were presented in a pseudo-random order using a Latin square design. At the end of each experiment, participants performed a control block in which the only task was the symbolic estimation of duration (30 s, 60 s or 90 s) of 12 trials (four trials per duration presented in random order) bounded by two red dots identical in every aspect to the ones used before. On each of these trials, only a fixation cross was displayed over a grey background.

### Statistical Analyses

All statistical analyses were carried out in the R programming language (R Core Team, 2017) and RStudio environment (RStudioTeam 2015), using the lme4^54^, betareg^55^ and lsmeans^56^ software packages. The logic of the statistical analyses reported in this study is described below for the different quantifications.

For duration estimates and for the reaction times (RTs) in the WM task, we used linear mixed effect (lme) models, which can be thought of as a generalization of linear regression models. In lme models, data are not aggregated so that statistics are made on all empirical observations. Additionally, and unlike repeated measures ANOVAs in which comparisons are made between averaged data (single-trial observations being lost), each observation was here taken into account and the inter-individual variability was considered as a random effect. This approach increases statistical power without over-fitting the data.

Separate regression models were fitted to the entire data set i.e. one for each participant. For all dependent variables (duration estimates in the duration task, and reaction times in WM), the initial lme model started with the mean component, the random effect and the dependent variable; we then incrementally added the predictor variables (e.g. duration length, WM load, task) to the initial model to see whether the model improved. The goodness-of-fits were assessed using the Akaike Information Criterion (AIC) and χ^2^ to compare different models. The significance of fixed factors can be assessed in two ways by the simplification of the regression model using the Akaike Information Criterion (AIC) or the likelihood ratio using Chi square (χ^2^). The AIC is a measure that optimizes model fit by taking into account the amount of explained variance as well as the degrees of freedom. This procedure ensures that the model achieves the best fit to the data with the minimum number of predictor variables. When two models are compared, the AIC provides information about whether the predictors added in the second model account for a significant amount of variance in the dependent variable. The best model corresponds to the minimal AIC. For instance, in the reported tables (e.g. Supp. Table 1), the list of models is provided along with their respective AIC. The model that best fit the data is the one with the minimal AIC. Consistent with this, the best models can also be found using Chi square. The best model using the likelihood measure is defined by a significant χ^2^ test (Pr (.Chisq)) comparing one model in the list to the next (e.g.: model 1 vs. 2, then model 2 vs. 3 and so on). The last comparison providing a significant effect points to the best model. The “ChisqChi” value corresponds to twice the difference of the log likelihood of the two models. Both AIC and Chisqu values are reported in Supp. Tables.

For the hit and false alarm rates in the WM task, we used a beta regression model in which the analysis of dependent variables was expressed as a ratio assuming values in a standard unit interval (0, 1)^57,58^. We used beta regression models because they can easily accommodate the asymmetry of heteroskedastic data such as hit and false alarm rates (acquired here in the WM task), whose variability increases around the mean but decreases towards the lower and upper limits of the standard unit^57,58^. Each model was built including two sub-models: a regression model for the mean, and a regression model for the variability. The later allowed information from the predictors to better estimate the non-normal distribution common in proportional data. The procedure for the beta regression models was similar to the one used in the lme so that after the initial model using the mean component, we added the precision component and applied the standard step-wise procedure. After completion of this procedure, we tested whether the inclusion of the precision component was justified by comparing the AIC of the initial model with the more complex model including both the mean and the precision components. This was tested with the likelihood ratio test.

Statistical significance between regression coefficients in the lme and beta regression models were directly drawn from the selected final model, and tested with t-tests and Wald tests yielding Z, respectively. The p-values in the lme models obtained from post-hoc analyses were adjusted using Tukey’s HSD. To avoid biases due to unbalanced data, tests of significance were made on the population marginal means^59^ estimated from linear models using the lsmeans R package^56^. The mean values and standard errors (SEs) reported in the Results section correspond to those extracted from the linear models. The values shown in the figures represent the arithmetic mean and standard errors (SEs) calculated from the empirical data, unless otherwise specified. For clarity, significance levels are sparingly used in Figures to highlight the main effects, but the full statistical effects are provided in the Results section and in Supplementary Tables. The specifics for the assessment of performance in the n-back WM and the duration estimation tasks are provided below.

#### n-back WM task

We assessed the performance on the n-back task using three dependent variables: Hit Rate (HR), False Alarm rate (FA) and Reaction Time (RT). The HR was the proportion of target letters participants accurately detected, and the FA was the proportion of non-target letters participants incorrectly responded to. A requirement of beta regression models is that response variables do not include the exact values 0 and/or 1^57^: proportions of 0 and 1 were thus converted to 1/(2N) and 1-1(2N), respectively, where N was the number of letters on which the proportion was based (Macmillan and Creelman 2004). RTs were computed from the onset of the displayed letter to the button press. RTs below 100 ms were discarded, representing ~3% of the total number of data points (i.e. 60 out of 2033). For each trial, the average RT was computed only for the accurately detected n-back targets. To address the effect of feedback and WM load on the n-back task performance, we fitted one beta regression model for HR separately from another one for FA; both HR and FA were dependent variables. A lme model was used for RT. Group (2 levels: *Feedback*, *No Feedback* (Fdbck and NoFdbck in Tables, respectively)) and WM load (4 levels: 0, 1, 2 and 3) were included as fixed effects. In the RT model, an intercept for Subjects (n = 48) was included as random mixed-effect.

#### Prospective duration estimation task

The 1^st^ and 3^rd^ quartiles were computed for the full range of time estimates, but also for the full range of response times (*i.e*., the time it took participants to give a time estimate using the number pad). The upper rejection boundary was computed as: 3^*rd*^ *Quartile* + 1.5 * (*IQR*) with IQR as the inter-quartile range. The lower rejection boundary was computed as: 1^*st*^ *Quartile* − 1.5 * (*IQR*). If a prospective judgment or a response time was above or below the respective upper or lower rejection boundaries, they were discarded from further analyses: ~8% of the total number of data points were excluded in both the Single-Task (*i.e*., 45 out of 552) and the Dual Task (*i.e*., 192 out of 2225).

The effect of attention on duration estimation was assessed using lme models. Task (2 levels: single-task (ST), dual-task (DT)) and Duration (3 levels: 30 s, 60 s, 90 s) were included as fixed-effects. Participants were considered as a random effect to control for their intra-class correlations. In order to analyze the general effect of attention on timing, data from the *Feedback* and *No Feedback* groups were pooled together given no paradigmatic difference for these conditions. The effect of WM load used one *lme* model for the *Feedback* and one *lme* model for the *No Feedback* groups. Tw by-subject random slopes were included, one for the effect of WM load and another one for the effect of Duration.

### Data accessibility

The datasets generated during the current study are available in the Working Memory & Duration repository in Open Science Framework osf.io/cg7ex.

## Electronic supplementary material

Supplementary Information

Example of a dual-task trial for the Feedback group
