# Publisher Correction: The effect of attention and working memory on the estimation of elapsed time

**DOI:** 10.1038/s41598-019-53224-z

**Published:** 2019-11-18

**Authors:** Ignacio Polti, Benoît Martin, Virginie van Wassenhove

**Affiliations:** 1 CEA, DRF/Joliot, NeuroSpin; INSERM, U992, Cognitive Neuroimaging Unit, Université Paris-Sud; Université Paris-Saclay, Gif/Yvette, France; 2 Kavli Institute for Systems Neuroscience, Centre for Neural Computation, The Egil and Pauline Braathen and Fred Kavli Centre for Cortical Microcircuits, NTNU, Norwegian University of Science and Technology, Trondheim, Norway

Correction to: *Scientific Reports* 10.1038/s41598-018-25119-y, published online 27 April 2018

A supplementary file, “Example of a dual-task trial for the Feedback group” was omitted from the original version of this Article. This has been corrected in the HTML version of the Article; the PDF version was correct at time of publication.

