# Supplementary material for: The effect of attention and working memory on the estimation of elapsed time
Source: Sci Rep. 2018 Apr 27;8:6690. doi: 10.1038/s41598-018-25119-y (PMC5923266; doi:10.1038/s41598-018-25119-y)
Supplement: Supplementary file 1 — Supplementary Information [file 41598_2018_25119_MOESM1_ESM.docx]

**The effect of attention and working memory on the estimation of elapsed time Ignacio Polti^1,2 *^, Benoît Martin^1^, Virginie van Wassenhove^1*^**

**SUPPLEMENTARY MATERIAL**

**SUPPLEMENTARY VIDEO**

**Example of a dual-task trial for the Feedback group.** The video illustrates a duration trial lasting 30 seconds and testing a 3-back working memory load. First, the trial starts with a screen indicating the n-back task, leaving the participant to start the trial when s/he wished for with “Pour continuer, appuyez sur la barre espace” (when ready, press the space bar). Once the participant initiates the trial, a red dot is displayed on the screen in order to mark the onset of the duration, hence that the participant should start timing. During this timing task, the participant also has to perform a 3-back WM task in which a letters are serially displayed on the screen. As we illustrate a trial tested in the Feedback group, we show a WM trial in which the participant is correct (“W” and “P” green colour changes) or incorrect (“C” red colour change). At the end of the sequence, another red dot signaled the end of the timing task and the participants has to evaluate how much time has passed between the two red dots. The screen displays the following instructions: “How much time has elapsed between the two red dots? Please use the number pad to give your answer in minutes and in seconds. Once you have finished, please press “enter””. The following trial follows, indicating which n-back task will take place in the following trials.

**SUPPLEMENTARY FIGURES**

**
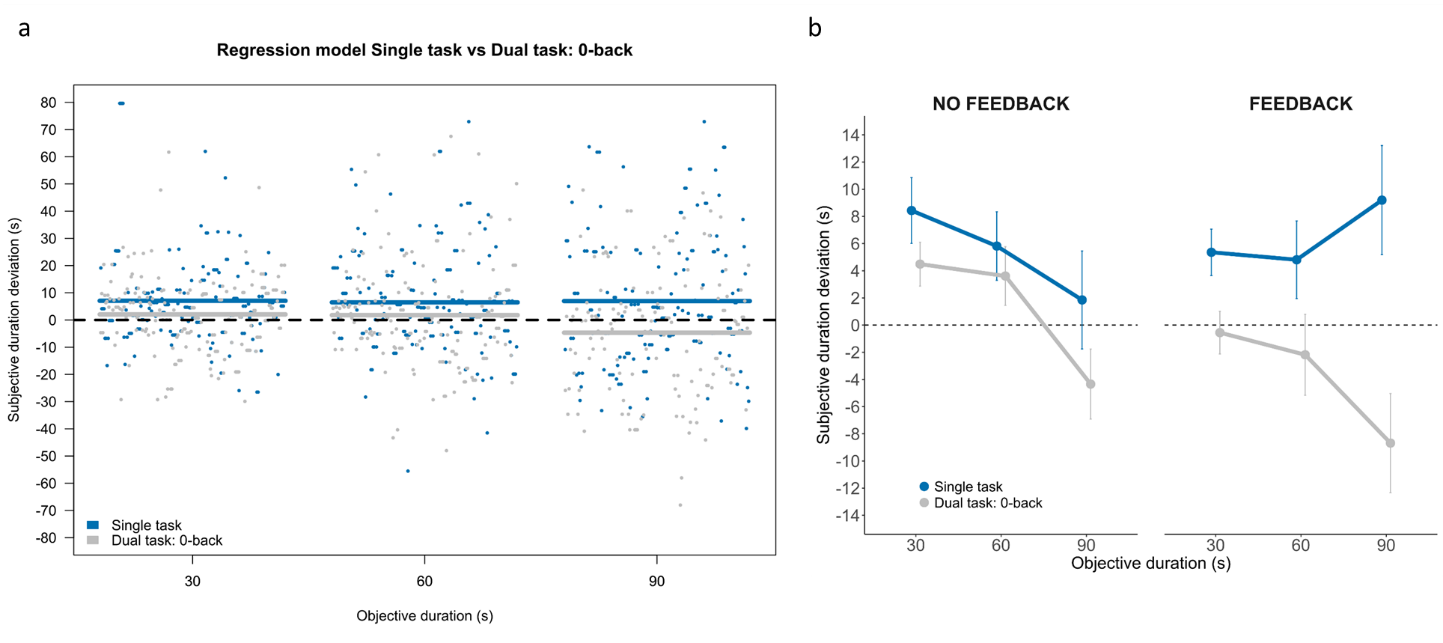
**

**SUPPLEMENTARY FIGURE 1: Panel a:** Regression model data for the single-task (blue) and 0-back dual-task (attention control, grey) conditions combining the *Feedback* and the *No Feedback* groups. Attending to time (single-task) significantly increased the estimation of duration equally for all three durations. In the 0-back dual-task condition, the estimation of duration was close to veridical or underestimated with compared to veridical condition. The difference between the single-task and 0-back dual-task was equivalent across the three tested durations (30 s, 60 s, 90 s). **Panel b:** Data illustrating the comparison between single-task and 0-back dual-task for the *Feedback* (right panel) and the *No Feedback* (left panel) groups. As can be seen, the general pattern, also reported in Figure 2a, was comparable in both groups with duration estimations in single-task being larger than in 0-back dual-task. The difference between the two groups was larger in the *Feedback* than in the *No Feedback* groups. However, no experimental manipulation differentiated the results for the single-task (blue; no WM task was used, no feedback was provided), thus suggesting random variability due to inter-individual variance. The main difference in the 0-back dual-task could be accounted for by attention or by changes in cognitive load induced by receiving feedback (see main text for discussion on this point). Error bars indicate s.e.m.


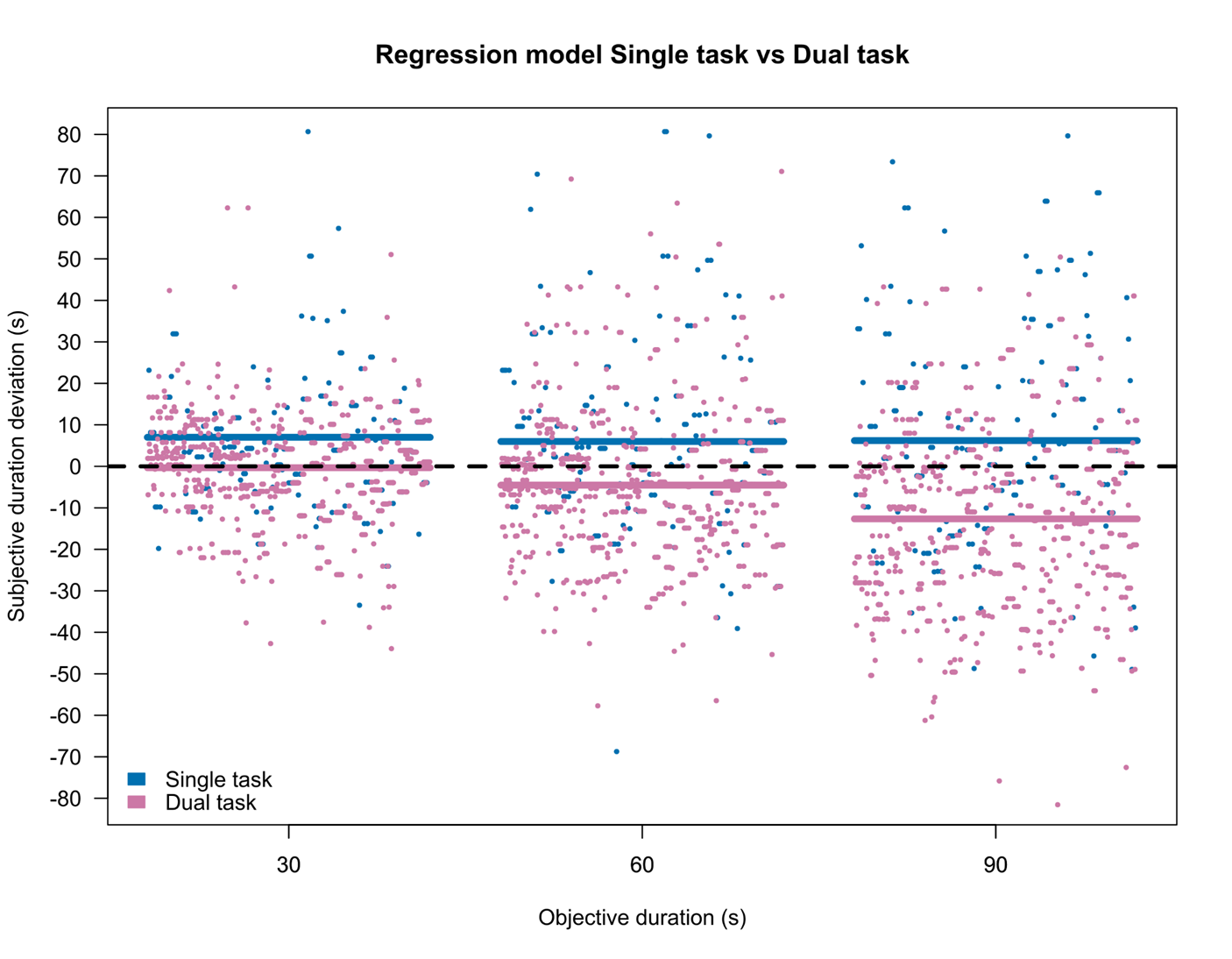


**SUPPLEMENTARY FIGURE 2:** Regression model data for the single-task (ST, blue) and dual-task (DT, all n, pink) conditions combining the Feedback and the No Feedback groups. Attending to time (ST) significantly increased the estimation of duration; attending away from time (DT) significantly decreased the estimation of duration. The difference between the ST and DT increased with duration, suggesting a possible scaling interference effect of WM load. Contrasts are reported in Supp. Table 3 and Supp. Table 5.

**
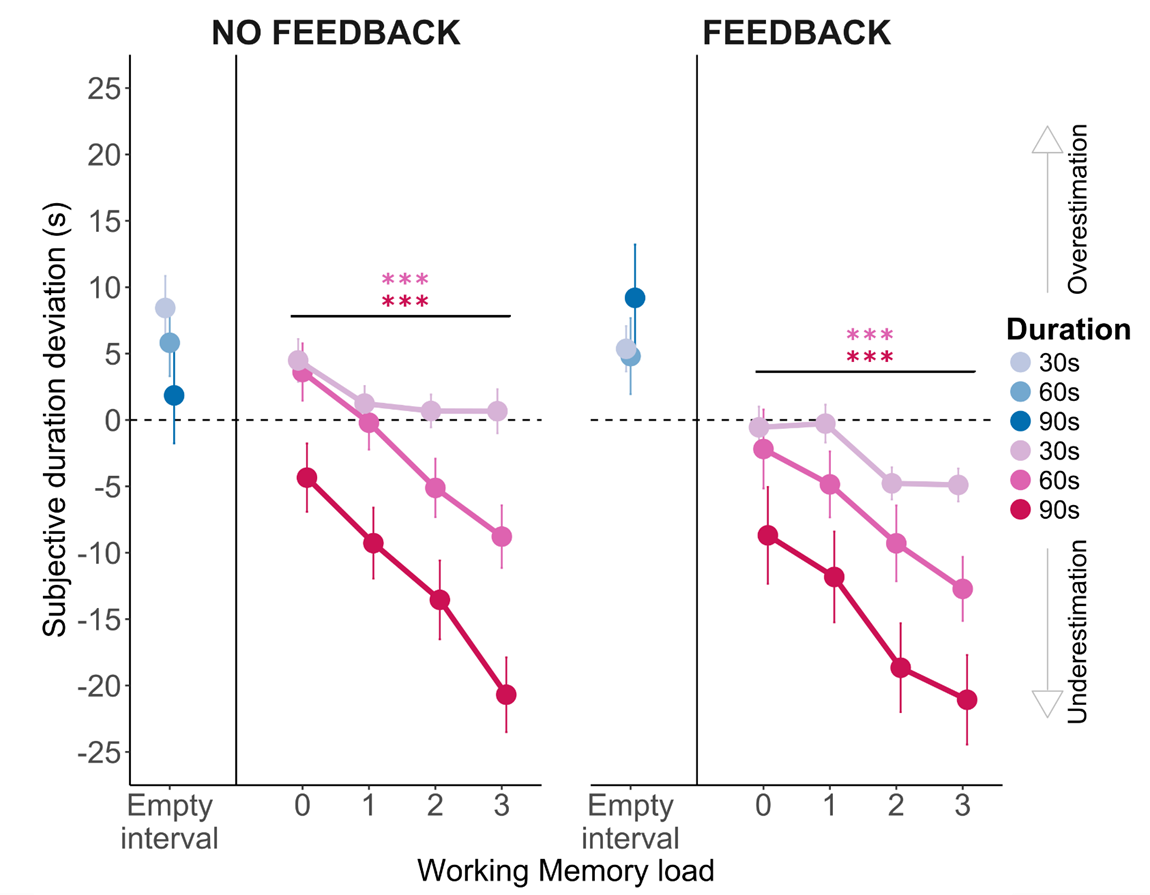
**

**SUPPLEMENTARY FIGURE 3: WM load incrementally affects duration estimation with or without feedback.** As in Figure 4a, subjective duration estimates were transformed to express the amount of over/under-estimation in the single-task (blue) and in the dual-task (pink) as a function of WM load conditions. Data for the *No Feedback* and the *Feedback* group are separately plotted (left and right panel, respectively). Hues are durations with lighter (darker) hues marking shorter (longer) durations. The dashed line represents the ideal observer so that positive values indicate a subjective overestimation of duration and negative values indicate a subjective underestimation of duration. Irrespective of feedback, durations were incrementally underestimated with WM load.

**TABLES**

**SUPPLEMENTARY TABLE 1: Beta regression models assessing performance (Hit Rates (HR) and False Alarm rates (FA)) in WM task.**

| **Hit Rates (HR)** | | | | | | |
| --- | --- | --- | --- | --- | --- | --- |
| **Mean models with logit link** | **Df** | **AIC** | **logLik** | **Chisq** | **Df** | **Pr(>Chisq)** |
| HRmodel_0: HR ~ 1 | 2 | -1159.468 | 581.73 | - | - | - |
| HRmodel_1: HR ~ WMload | 5 | -1995.080 | 982.54 | 801.61 | 3 | <2.2e-16 *** |
| HRmodel 2: HR ~ WMload + Group | 6 | -2043.845 | 1027.92 | 90.765 | 1 | <2.2e-16 *** |
| HRmodel 3: HR ~ WMload * Group | 9 | -2055.904 | 1037.0 | 18.06 | 3 | 0.0004276 *** |
| **Precision models with log link** | **Df** | **AIC** | **logLik** | **Chisq** | **Df** | **Pr(>Chisq)** |
| HRmodel_phi_0 = HR ~ 1 \| 1 | 2 | -1159.468 | 581.73 | - | - | - |
| HRmodel_phi_1 = HR ~ 1 \| WMload | 5 | -1282.585 | 646.29 | 129.12 | 3 | <2.2e-16 *** |
| HRmodel_phi_2 = HR ~ 1 \| WMload + Group | 6 | -1280.603 | 646.30 | 0.0189 | 1 | 0.8907 |
| HRmodel_phi_3 = HR ~ 1 \| WMload * Group | 9 | -1315.183 | 666.59 | 40.598 | 4 | 3255e-08 *** |
| **Mean + precision models** | **Df** | **AIC** | **logLik** | **Chisq** | **Df** | **Pr(>Chisq)** |
| HRmodel_comb = HR ~ WMload * Group \| WMload * Group | 16 | -2458.972 | 1245.5 | 417.07 | 7 | <2.2e-16 *** |
| **False Alarm rate (FA)** | | | | | | |
| **Mean models with logit link** | **Df** | **AIC** | **logLik** | **Chisq** | **Df** | **Pr(>Chisq)** |
| FAmodel_0: FA ~ 1 | 2 | -11246.25 | 5803.6 | - | - | - |
| FAmodel_1: FA ~ WMload | 5 | -11597.25 | 5803.6 | 357 | 3 | <2.2e-16 *** |
| FAmodel 2: FA ~ WMload + Group | 6 | -11648.82 | 5830.4 | 53.568 | 1 | <2.498e-13 *** |
| FAmodel 3: FA ~ WMload * Group | 9 | -11652.51 | 5835.3 | 9.6927 | 3 | 0.02137 * |
| **Precision models with log link** | **Df** | **AIC** | **logLik** | **Chisq** | **Df** | **Pr(>Chisq)** |
| FAmodel_phi_0 = FA ~ 1 \| 1 | 2 | -11246.25 | 5625.1 | - | - | - |
| FAmodel_phi_1 = FA ~ 1 \| WMload | 5 | -11319.23 | 5664.6 | 78.984 | 3 | <2.2e-16 *** |
| FAmodel_phi_2 = FA ~ 1 \| WMload + Group | 6 | -11323.42 | 5667.7 | 6.186 | 1 | 0.01288 * |
| FAmodel_phi_3 = FA ~ 1 \| WMload * Group | 9 | -11334.57 | 5676.3 | 23.34 | 4 | 0.0001083 *** |
| **Mean + precision models** | **Df** | **AIC** | **logLik** | **Chisq** | **Df** | **Pr(>Chisq)** |
| FAmodel_comb = FA ~ WMload * Group\| WMload * Group | 16 | -12025.66 | 6028.8 | 387.15 | 7 | <2.2e-16 *** |

**SUPPLEMENTARY TABLE 2: Differences between *Feedback* and *No Feedback* groups in the WM task.** Hit Rate (HR) and False Alarm rate (FA) post-hoc contrasts from beta regression models. Reaction Times (RT) post-hoc contrasts from *lme* model. All data are provided in Figure 1c.

|  | | **Hit Rate (HR)** | **False Alarm (FA)** | **Reaction Time (RT)** |
| --- | --- | --- | --- | --- |
|  |  | **Main effect of Group** | | |
| **FEEDBACK *vs.* NO FEEDBACK** | **0-back** | contrast coefficient: -0.06244  z value = -7.661, *** | contrast coefficient: -0.11226  z value = -2.317, n. s. | contrast coefficient: -61.01746  t value = -4.342, *** |
|  | **1-back** | contrast coefficient: -0.02638  z value = -2.975, ** | contrast coefficient: -0.11226  z value = -2.317, n. s. | contrast coefficient: -92.22434  t value = -6.582, *** |
|  | **2-back** | contrast coefficient: -0.06890  z value = -3.842, ** | contrast coefficient: -0.18717  z value = -3.976, *** | contrast coefficient: -137.15961  t value = -9.785, *** |
|  | **3-back** | contrast coefficient: -0.12491  z value = -6.078, *** | contrast coefficient: -0.28340  z value = -4.640, *** | contrast coefficient: -168.78043  t value = -11.931, *** |
|  | | **Main effect of WM load** | | |
| **NO FEEDBACK** | **0-back *vs.* 1-back** | contrast coefficient: 0.04422  z value = 5.914, *** | contrast coefficient: -0.005609  z value = -0.121, n. s. | contrast coefficient: -32.816257  t value = -5.668, *** |
|  | **1-back *vs.* 2-back** | contrast coefficient: 0.15349  z value = 11.187, *** | contrast coefficient: 0.521595  z value = 9.531, *** | contrast coefficient: -79.639722  t value = -13.740, *** |
|  | **2-back *vs.* 3-back** | contrast coefficient 0.11452  z value = 5.823, *** | contrast coefficient 0.784992  z value = 14.034, *** | contrast coefficient -40.051693  t value = -6.722, *** |
| **FEEDBACK** | **0-back *vs.* 1-back** | contrast coefficient: 0.00815  z value = 0.864, n. s. | contrast coefficient: 0.06930  z value = 1.406, n.s. | contrast coefficient: -1.609377  t value = -0.278, n. s. |
|  | **1-back *vs.* 2-back** | contrast coefficient: 0.19601  z value = 13.460, *** | contrast coefficient: 0.69273  z value = 12.476, *** | contrast coefficient: -34.704446  t value = -6.101, *** |
|  | **2-back *vs.* 3-back** | contrast coefficient: 0.14053  z value = 9.021, *** | contrast coefficient 0.91849  z value = 16.762, *** | contrast coefficient: -8.430877  t value = -1.443, n. s. |
|  | | **Interaction effect WM load * Group** | | |
| **FEEDBACK – NO FEEDBACK** | **0-back *vs.* 1-back** | contrast coefficient: -0.03606  z value: -2.994, ** | contrast coefficient: -0.00127  z value: -1.169, ns | contrast coefficient: 31.20688  t value: 3.815, *** |
|  | **0-back *vs.* 2-back** | contrast coefficient: 0.00645  z value: 0.328, n. s. | contrast coefficient: -0.00623  z value: -3.270, ** | contrast coefficient: 76.14216  t value: 9.295, *** |
|  | **0-back *vs.* 3-back** | contrast coefficient: 0.06246  z value: 2.825, significance ** | contrast coefficient: -0.00995  z value: -4.095, *** | contrast coefficient: 107.76297  t value: 12.804, significance *** |
|  | **1-back *vs.* 2-back** | contrast coefficient: 0.04252  z value: 2.125, significance * | contrast coefficient: -0.00495  z value: -2.597, ** | contrast coefficient: 44.93528  t value: 5.533, significance *** |
|  | **1-back *vs.* 3-back** | contrast coefficient: 0.09853  z value: 4.402, significance *** | contrast coefficient: -0.00868  z value: -3.567, ** | contrast coefficient: 76.55609  t value: 9.172, significance *** |
|  | **2-back *vs.* 3-back** | contrast coefficient: 0.05601  value: 2.053, significance * | contrast coefficient: -0.00372  z value: -1.288, n. s. | contrast coefficient: 31.62082  t value: 3.790, significance *** |

**SUPPLEMENTARY TABLE 3: Mixed linear regression models.** The question addressed by each model is provided in the first line of each sub-table along with the Figure in which associated data can be visualized.

| **Reaction Times in WM task (Figure 1c, right panel)** | | | | | | |
| --- | --- | --- | --- | --- | --- | --- |
|  | **Df** | **AIC** | **logLik** | **Chisq** | **Df** | **Pr(>Chisq)** |
| RTmodel_0: RT ~ 1 + (1 \| Subject) | 3 | 22683.53 | -11339 | - | - | - |
| RTmodel_1: RT ~ WM load + (1 \| Subject) | 6 | 22112.28 | -11050 | 577.25 | 3 | <2.2e-16 *** |
| RTmodel_2: RT ~ WM load + Group + (1 \| Subject) | 7 | 22068.96 | -11028 | 45.319 | 1 | 1.674e-11 *** |
| RTmodel_3: RT ~ WM load * Group + (1 \| Subject) | 10 | 21889.88 | -10935 | 185.09 | 3 | <2.2e-16 *** |
| **Effect of attention on subjective duration deviation (Δt) in single-task and 0-back dual task (Figure 2a; Supp. Fig. 1a )** | | | | | | |
| ST*vs*.DT0-back_model_0: Δt ~ 1 + (1 \| Subject) | 3 | 8961.652 | -4542.6 | - | - | - |
| ST*vs*.DT0-back_model_1: Δt ~ Task + (1 \| Subject) | 4 | 8934.233 | -4463.1 | 29.419 | 1 | 5.831e-08 *** |
| ST*vs*.DT0-back_model_2: Δt ~ Task + Duration + (1 \| Subject) | 6 | 8932.967 | -4460.5 | 5.2665 | 2 | 0.07185 n. s. |
| ST*vs*.DT0-back_model_3: Δt ~ Task * Duration + (1 \| Subject) | 8 | 8930.645 | - 4457.3 | 6.3223 | 2 | 0.04238 * |
| **Single task vs Dual task: 0-back Coefficient of Variation (CV). Mixed linear regression (Figure 2a inset)** | | | | | | |
| CV_STvs.DT0-back_model_0: CV ~ 1 + (1 \| Subject) | 3 | -281.647 | 143.82 | - | - | - |
| CV_STvs.DT0-back_model_1: CV ~ Duration + (1 \| Subject) | 5 | -282.139 | 146.07 | 4.4926 | 2 | 0.1058 n. s. |
| CV_STvs.DT0-back_model_1b: CV ~ Task + (1 \| Subject) | 4 | -280.295 | 144.15 | 0.6481 | 1 | 0.4208 n. s. |
| **Subjective duration deviation (Δt) in Single *vs.* Dual Task. Mixed linear regression (Fig. 2b; Supp. Fig. 2)** | | | | | | |
|  | **Df** | **AIC** | **logLik** | **Chisq** | **Df** | **Pr(>Chisq)** |
| Dev_S*vs*.Dtask_model_0: Δt ~ 1 + (1 \| Subject) | 3 | 3921.950 | -11253 | - | - | - |
| Dev_S*vs*.Dtask_model_1: Δt ~ Task + (1 \| Subject) | 4 | 22358.67 | -11175 | 155.28 | 1 | <2.2e-16 *** |
| Dev_S*vs*.Dtask_model_2: Δt ~ Task + Duration + (1 \| Subject) | 6 | 22242.14 | -11115 | 120.53 | 2 | <2.2e-16 *** |
| Dev_S*vs*.Dtask_model_3: Δt ~ Task * Duration + (1 \| Subject) | 8 | 22218.84 | -11101 | 27.306 | 2 | 1.176e-06 *** |
| **Subjective duration variance (σ²). Mixed linear regression in Single *vs.* Dual Task (Fig. 2c)** | | | | | | |
| Var_S*vs*.Dtask_model_0: σ² ~ 1 + (1 \| Subject) | 3 | 3886.642 | -1958.0 | - | - | - |
| Var_S*vs*.Dtask_model_1: σ² ~ Duration + (1 \| Subject) | 5 | 3854.956 | -1922.5 | 70.994 | 2 | 3.837e-16 *** |
| Var_S*vs*.Dtask_model_2: σ² ~ Duration + Task + (1 \| Subject) | 6 | 3845.502 | -1916.8 | 11.454 | 1 | 0.0007132 *** |
| Var_S*vs*.Dtask_model_3: σ² ~ Duration * Task + (1 \| Subject) | 8 | 3828.397 | -1906.2 | 21.105 | 2 | 2.613e-05 *** |
| **Effect of Task on Coefficient of Variation (CV). Mixed linear regression (Fig. 2d)** | | | | | | |
| CV_Svs.Dtask_model_0: CV ~ 1 + (1 \| Subject) | 3 | -351.223 | 178.61 | - | - | - |
| CV_Svs.Dtask_model_1: CV ~ Task + (1 \| Subject) | 4 | -378.571 | 193.29 | 29.348 | 1 | 6.048e-08 *** |
| **WM effect on subjective duration deviation (Δt) (Fig. 3a)** | | | | | | |
| WM_model_0: Δt ~ 1 + (1 \| Subject) | 3 | 17546.20 | -8770.1 | - | - | - |
| WM_model_1: Δt ~ WM load + (1 \| Subject) | 6 | 17427.91 | -8708.0 | 124.29 | 3 | <2.2e-16 *** |
| WM_model_2: Δt ~ WM load + Duration + (1 \| Subject) | 8 | 17250.33 | -8617.2 | 181.58 | 2 | <2.2e-16 *** |
| WM_model_3: Δt ~ WM load * Duration + (1 \| Subject) | 14 | 17235.59 | - 8603.8 | 26.736 | 6 | 0.0001623 *** |
| **WM effect on subjective duration deviation (Δt), *Feedback g*roup (Supp. Figure 3)** | | | | | | |
|  | **Df** | **AIC** | **logLik** | **Chisq** | **Df** | **Pr(>Chisq)** |
| WM_model_Fdbck_0: Δt ~ 1 + (1 \| Subject) | 3 | 8993.553 | -4493.8 | - | - | - |
| WM_model_Fdbck_1: Δt ~ WM load + (1 \| Subject) | 6 | 8936.109 | -4462.1 | 63.444 | 3 | 1.079e-13 *** |
| WM_model_Fdbck_2: Δt ~ WM load + Duration + (1 \| Subject) | 8 | 8863.054 | -4423.5 | 77.055 | 2 | <2.2e-16 *** |
| **WM effect on subjective duration deviation (Δt), *No Feedback g*roup (Supp. Figure 3)** | | | | | | |
| WM_model_NoFdbck_0: Δt ~ 1 + (1 \| Subject) | 3 | 8551.581 | -4272.8 | - | - | - |
| WM_model_NoFdbck_1: Δt ~ WM load + (1 \| Subject) | 6 | 8494.037 | -4241 | 63.544 | 3 | 1.027e-13 *** |
| WM_model_NoFdbck_2: Δt ~ WM load + Duration + (1 \| Subject) | 8 | 8389.775 | -4186.9 | 108.26 | 2 | <2.2e-16 *** |
| WM_model_NoFdbck_3: Δt ~ WM load * Duration + (1 \| Subject) | 14 | 8378.529 | -4175.3 | 23.246 | 6 | 0.0007182 *** |
| **Effect of WM load on Coefficient of Variation (CV), Mixed linear regression (Figure 3c)** | | | | | | |
| CV_WMload_model_0: CV ~ 1 + (1 \| Subject) | 3 | -466.064 | 236.03 | - | - | - |
| CV_WMload_model_1: CV ~ WM load + (1 \| Subject) | 6 | -476.114 | 244.06 | 16.05 | 3 | 0.001108 ** |

**SUPPLEMENTARY TABLE 4: Linear mixed effect model post-hoc contrasts assessing the effect of attention on subjective duration in the single task *vs*. the 0-back dual-task.** Data in Figure 2a and Supp. Fig. 1.

| **Main effect of Task** | | |
| --- | --- | --- |
| **SINGLE TASK *vs.***  **0-BACK DUAL TASK** | **30 s** | contrast coefficient: 4.992350  t value = 2.404, * |
|  | **60 s** | contrast coefficient: 4.692745  t value = 2.191, * |
|  | **90 s** | contrast coefficient: 11.606930  t value = 5.182, *** |
| **Main effect of Duration** | | |
| **SINGLE-TASK** | **30s *vs*. 60s** | contrast coefficient: 0.56786460  t value = 0.269, n. s. |
|  | **30s *vs*. 90s** | contrast coefficient: 0.09731831  t value = 0.046, n. s. |
|  | **60s *vs*. 90s** | contrast coefficient: -0.47054629  t value = -0.219, n. s. |
| **0-BACK DUAL TASK** | **30s *vs*. 60s** | contrast coefficient: 0.26825898  t value =0.128, n. s. |
|  | **30s *vs*. 90s** | contrast coefficient: 6.71189824  t value = 3.077, ** |
|  | **60s *vs*. 90s** | contrast coefficient: 6.44363926  t value = 2.894, * |

**SUPPLEMENTARY TABLE 5: Subjective duration estimation as a function of single and dual-tasks.** Post-hoc contrasts of Linear mixed effect model. Data in Figure 2b and Supp. Fig. 2.

| **Main effect of Task** | | |
| --- | --- | --- |
| **SINGLE TASK *vs.* DUAL TASK** | **30 s** | contrast coefficient: 7.319659  t value = 4.678, *** |
|  | **60 s** | contrast coefficient: 10.526908  t value = 6.615, *** |
|  | **90 s** | contrast coefficient: 18.853225  t value = 11.548, *** |
| **Main effect of Duration** | | |
| **DUAL TASK** | **30s *vs*. 60s** | contrast coefficient: 4.2107511  t value = 4.239, *** |
|  | **30s *vs*. 90s** | contrast coefficient: 12.3333878  t value = 12.192, *** |
|  | **60s *vs*. 90s** | contrast coefficient: 8.1226368  t value = 7.936, *** |
| **Interaction effect Task * Duration** | | |
| **SINGLE TASK *vs*. DUAL** | **30s *vs*. 60s** | contrast coefficient: -3.207248  t value = -1.430, n. s. |
|  | **30s *vs*. 90s** | contrast coefficient: -11.533566  t value = -5.103, *** |
|  | **60s *vs*. 90s** | contrast coefficient: -8.326317  t value = -3.656, significance ** |

**SUPPLEMENTARY TABLE 6: Variance of subjective duration estimates in single-task and in dual-task condition.** Linear mixed effect model post-hoc contrasts. Data in Figure 2c.

| **Main effect of Duration** | | |
| --- | --- | --- |
| **SINGLE TASK** | **60 s *vs.* 30 s** | contrast coefficient: 120.69546  t value = 2.475, * |
|  | **90 s *vs.* 30 s** | contrast coefficient: 251.45038  t value = 5.125, *** |
|  | **90 s *vs.* 60 s** | contrast coefficient: 130.75492  t value = 2.652, * |
| **DUAL TASK** | **60 s *vs.* 30 s** | contrast coefficient: 181.63153  t value = 3.829, ***- |
|  | **90 s *vs.* 30 s** | contrast coefficient: 272.33354  t value = 5.741, *** |
|  | **90 s *vs.* 60 s** | contrast coefficient: 90.70201  t value = 1.912, n. s. |

**SUPPLEMENTARY TABLE 7: Effect of single- and dual-task on the coefficient of variation.** Mixed linear regression post-hoc contrasts**.** Data in Figure 2d.

| **Main effect of Task** | | |
| --- | --- | --- |
| **SINGLE TASK**  ***vs.***  **DUAL-TASK** | **30 s** | contrast coefficient: -0.07025887  t value = -3.058, ** |
|  | **60 s** | contrast coefficient: -0.08848000  t value = -3.826, ** |
|  | **90 s** | contrast coefficient: -0.06824674  t value = -2.932, ** |

**SUPPLEMENTARY TABLE 8: Effect of WM load on the subjective estimation of duration.** Linear mixed effect model post-hoc contrasts**.** Data in Figure 3a.

| **Main effect of WM load** | | |
| --- | --- | --- |
| **30 s** | **WM load = 0 - WM load = 1** | contrast coefficient: 1.78870617  t value = 1.067, n. s. |
|  | **WM load = 0 - WM load = 2** | contrast coefficient: 4.30797224  t value = 2.560, n. s. |
|  | **WM load = 0 - WM load = 3** | contrast coefficient: 4.39296285  t value = 2.594, * |
|  | **WM load = 1 - WM load = 2** | contrast coefficient: 2.51926607  t value = 1.494, n. s. |
|  | **WM load = 1 - WM load = 3** | contrast coefficient: 2.60425668  t value 1.536, n. s. |
|  | **WM load = 2 - WM load = 3** | contrast coefficient: 0.08499061  t value = 0.050, n. s. |
| **60 s** | **WM load = 0 - WM load = 1** | contrast coefficient: 4.30973553  t value = 2.467, n. s. |
|  | **WM load = 0 - WM load = 2** | contrast coefficient: 9.12220332  t value = 5.253, *** |
|  | **WM load = 0 - WM load = 3** | contrast coefficient: 12.31659185  t value = 7.093, *** |
|  | **WM load = 1 - WM load = 2** | contrast coefficient: 4.81246778  t value = 2.785, * |
|  | **WM load = 1 - WM load = 3** | contrast coefficient: 8.00685631  t value = 4.621, *** |
|  | **WM load = 2 - WM load = 3** | contrast coefficient: 3.19438853  t value = 1.855, n. s. |
| **90 s** | **WM load = 0 - WM load = 1** | contrast coefficient: 4.07337844  t value = 2.223, n. s. |
|  | **WM load = 0 - WM load = 2** | contrast coefficient: 10.89606425  t value = 6.017, *** |
|  | **WM load = 0 - WM load = 3** | contrast coefficient: 15.92716937  t value = 8.792, *** |
|  | **WM load = 1 - WM load = 2** | contrast coefficient: 6.82268582  t value = 3.852, *** |
|  | **WM load = 1 - WM load = 3** | contrast coefficient: 11.85379093  t value = 6.689, *** |
|  | **WM load = 2 - WM load = 3** | contrast coefficient: 5.03110512  t value = 2.877, * |
| **Main effect of Duration** | | |
| **0-BACK** | **30s *vs*. 60s** | contrast coefficient: 0.4092973  t value = 0.239, n. s. |
|  | **30s *vs*. 90s** | contrast coefficient: 6.8287113  t value = 3.842, *** |
|  | **60s *vs*. 90s** | contrast coefficient 6.4194140  t value = 3.537, ** |
| **1-BACK** | **30s *vs*. 60s** | contrast coefficient 2.9303267  t value = 1.715, n. s. |
|  | **30s *vs*. 90s** | contrast coefficient: 9.1133836  t value = 5.243, *** |
|  | **60s *vs*. 90s** | contrast coefficient: 6.1830569  t value = 3.498, ** |
| **2-BACK** | **30s *vs*. 60s** | contrast coefficient: 5.2235284  t value = 3.063, ** |
|  | **30s *vs*. 90s** | contrast coefficient: 13.4168033  t value = 7.794, *** |
|  | **60s *vs*. 90s** | contrast coefficient: 8.3604964  t value = 4.726, *** |
| **3-BACK** | **30s *vs*. 60s** | contrast coefficient: 8.3329263  t value = 4.847, *** |
|  | **30s *vs*. 90s** | contrast coefficient: 18.3629178  t value = 10.612, *** |
|  | **60s *vs*. 90s** | contrast coefficient: 10.0299915  t value = 5.770, *** |
| **Interaction effect WM load * Duration** | | |
| **30 s - 60 s** | **0-back *vs*. 1-back** | contrast coefficient: -2.5210294  t value = -1.041, n. s. |
|  | **0-back *vs*. 2-back** | contrast coefficient: -4.8142311  t value = -1.991, * |
|  | **0-back *vs*. 3-back** | contrast coefficient: -7.9236290  t value = -3.263, ** |
|  | **1-back *vs*. 2-back** | contrast coefficient: -2.2932017  t value = -0.950, n. s. |
|  | **1-back *vs*. 3-back** | contrast coefficient: -5.4025996  t value = -2.229, * |
|  | **2-back *vs*. 3-back** | contrast coefficient: -3.1093979  t value = -1.284, n. s. |
| **30 s - 90 s** | **0-back *vs*. 1-back** | contrast coefficient: -2.2846723  t value = -0.920, n. s. |
|  | **0-back *vs*. 2-back** | contrast coefficient: -6.5880920  t value = -2.665, *** |
|  | **0-back *vs*. 3-back** | contrast coefficient: -11.5342065  t value = -4.653, *** |
|  | **1-back *vs*. 2-back** | contrast coefficient: -4.3034197  t value = -1.760, n. s. |
|  | **1-back *vs*. 3-back** | contrast coefficient: -9.2495343  t value = -3.773, *** |
|  | **2-back *vs*. 3-back** | contrast coefficient: -4.9461145  t value = -2.027, * |
| **60 s - 90 s** | **0-back *vs*. 1-back** | contrast coefficient: 0.2363571  t value = 0.093, n. s. |
|  | **0-back *vs*. 2-back** | contrast coefficient: -1.7738609  t value = -0.707, n. s. |
|  | **0-back *vs*. 3-back** | contrast coefficient: -3.6105775  t value = -1.437, n. s. |
|  | **1-back *vs*. 2-back** | contrast coefficient: -2.0102180  t value = -0.812, n. s. |
|  | **1-back *vs*. 3-back** | contrast coefficient: -3.8469346  t value = -1.552, n. s. |
|  | **2-back *vs*. 3-back** | contrast coefficient: -1.8367166  t value = -0.748, n. s. |

**SUPPLEMENTARY Table 9: Effect of WM load on coefficient of variation in dual-task.** Mixed linear regression post-hoc contrasts. Data in Figure 3c.

| **Main effect of WM load** | |
| --- | --- |
| **0-back *vs.* 1-back** | contrast coefficient: -0.002022898  t value = -0.115, n. s. |
| **0-back *vs.* 2-back** | contrast coefficient: -0.041062479  t value = -2.343, n. s. |
| **0-back *vs.* 3-back** | contrast coefficient: -0.057869357  t value = -3.300, ** |
| **1-back *vs.* 2-back** | contrast coefficient: -0.039039581  t value = -2.240, n. s. |
| **1-back *vs.* 3-back** | contrast coefficient: -0.055846459  t value = -3.203, ** |
| **2-back *vs.* 3-back** | contrast coefficient: -0.016806878  t value = -0.970, n. s. |

**SUPPLEMENTARY TABLE 10: Subjective duration deviation (Δt) as a function of WM load in *Feedback* and *No Feedback* groups.** Linear mixed effect model post-hoc contrasts. Data in Supp Fig 3.

| **Main effect of WM load** | | | | |
| --- | --- | --- | --- | --- |
| **FEEDBACK** | **30 s** | **WM load = 0 - WM load = 1** | | contrast coefficient: 0.2144352  t value = 0.087, n. s. |
|  |  | **WM load = 0 - WM load = 2** | | contrast coefficient: 4.5461319  t value = 1.833, n. s. |
|  |  | **WM load = 0 - WM load = 3** | | contrast coefficient: 5.4812505  t value = 2.230, n. s. |
|  |  | **WM load = 1 - WM load = 2** | | contrast coefficient: 4.3316967  t value = 1.746, n. s. |
|  |  | **WM load = 1 - WM load = 3** | | contrast coefficient: 5.2668152  t value = 2.143, n. s. |
|  |  | **WM load = 2 - WM load = 3** | | contrast coefficient: 0.9351186  t value = 0.376, n. s. |
|  | **60 s** | **WM load = 0 - WM load = 1** | | contrast coefficient: 3.9133205  t value = 1.519, n. s. |
|  |  | **WM load = 0 - WM load = 2** | | contrast coefficient: 8.5793824  t value = 3.337, ** |
|  |  | **WM load = 0 - WM load = 3** | | contrast coefficient: 11.8944056  t value = 4.701, *** |
|  |  | **WM load = 1 - WM load = 2** | | contrast coefficient: 4.6660619  t value = 1.844, n. s. |
|  |  | **WM load = 1 - WM load = 3** | | contrast coefficient: 7.9810851  t value = 3.207, ** |
|  |  | **WM load = 2 - WM load = 3** | | contrast coefficient: 3.3150232  t value = 1.336, n. s. |
|  | **90 s** | **WM load = 0 - WM load = 1** | | contrast coefficient: 1.7957461  t value = 0.657, n. s. |
|  |  | **WM load = 0 - WM load = 2** | | contrast coefficient: 11.0468017  t value = 4.144, *** |
|  |  | **WM load = 0 - WM load = 3** | | contrast coefficient: 13.8990768  t value = 5.196, *** |
|  |  | **WM load = 1 - WM load = 2** | | contrast coefficient: 9.2510556  t value = 3.585, ** |
|  |  | **WM load = 1 - WM load = 3** | | contrast coefficient: 12.1033307  t value = 4.675, *** |
|  |  | **WM load = 2 - WM load = 3** | | contrast coefficient: 2.8522751  t value = 1.134, n. s. |
| **NO FEEDBACK** | **30 s** | **WM load = 0 - WM load = 1** | | contrast coefficient: 3.4115291  t value = 1.503, n. s. |
|  |  | **WM load = 0 - WM load = 2** | | contrast coefficient: 4.0857406  t value = 1.805, n. s. |
|  |  | **WM load = 0 - WM load = 3** | | contrast coefficient: 3.1896274  t value = 1.379, n. s. |
|  |  | **WM load = 1 - WM load = 2** | | contrast coefficient: 0.6742115  t value = 0.297, n. s. |
|  |  | **WM load = 1 - WM load = 3** | | contrast coefficient: -0.2219017  t value = - 0.096, n. s. |
|  |  | **WM load = 2 - WM load = 3** | | contrast coefficient: - 0.8961132  t value = - 0.387, n. s. |
|  | **60 s** | **WM load = 0 - WM load = 1** | | contrast coefficient: 4.6481352  t value = 1.981, n. s. |
|  |  | **WM load = 0 - WM load = 2** | | contrast coefficient: 9.6165152  t value = 4.138, *** |
|  |  | **WM load = 0 - WM load = 3** | | contrast coefficient: 12.6198817  t value = 5.297, *** |
|  |  | **WM load = 1 - WM load = 2** | | contrast coefficient: 4.9683800  t value = 2.125, n. s. |
|  |  | **WM load = 1 - WM load = 3** | | contrast coefficient: 7.9717465  t value = 3.328, ** |
|  |  | **WM load = 2 - WM load = 3** | | contrast coefficient: 3.0033665  t value = 1.265, n. s. |
|  | **90 s** | **WM load = 0 - WM load = 1** | | contrast coefficient: 6.2645429  t value = 2.575, * |
|  |  | **WM load = 0 - WM load = 2** | | contrast coefficient: 10.6087405  t value = 4.344, *** |
|  |  | **WM load = 0 - WM load = 3** | | contrast coefficient: 18.0304336  t value = 7.406, *** |
|  |  | **WM load = 1 - WM load = 2** | | contrast coefficient: 4.3441976  t value = 1.802, n. s. |
|  |  | **WM load = 1 - WM load = 3** | | contrast coefficient: 11.7658906  t value = 4.893, *** |
|  |  | **WM load = 2 - WM load = 3** | | contrast coefficient: 7.4216931  t value = 3.078, * |
| **Main effect of Duration** | | | | |
| **FEEDBACK** | **0-back** | | **30s *vs.* 60s** | contrast coefficient: 1.017315  t value = 0.401, n. s. |
|  |  |  | **30s *vs.* 90s** | contrast coefficient: 7.170500  t value = 2.714, * |
|  |  |  | **60s *vs.* 90s** | contrast coefficient: 6.153185  t value = 2.261, n. s. |
|  | **1-back** | | **30s *vs.* 60s** | contrast coefficient: 4.716200  t value = 1.890, n. s. |
|  |  |  | **30s *vs*. 90s** | contrast coefficient: 8.751810  t value = 3.425, ** |
|  |  |  | **60s *vs*. 90s** | contrast coefficient: 4.035610  t value = 1.554, n. s. |
|  | **2-back** | | **30s *vs*. 60s** | contrast coefficient: 5.050566  t value = 2.008, n. s. |
|  |  |  | **30s *vs*. 90s** | contrast coefficient: 13.671169  t value = 5.450, *** |
|  |  |  | **60s *vs*. 90s** | contrast coefficient: 8.620604  t value = 3.426, ** |
|  | **3-back** | | **30s *vs*. 60s** | contrast coefficient: 7.430470  t value = 3.031, ** |
|  |  |  | **30s *vs*. 90s** | contrast coefficient: 15.588326  t value = 6.250, *** |
|  |  |  | **60s *vs*. 90s** | contrast coefficient: 8.157856  t value = 3.288, ** |
| **NO FEEDBACK** | **0-back** | | **30s *vs*. 60s** | contrast coefficient: -0.1465113  t value = -0.064, n. s. |
|  |  |  | **30s *vs*. 90s** | contrast coefficient: 6.5505515  t value = 2.763, * |
|  |  |  | **60s *vs*. 90s** | contrast coefficient: 6.6970628  t value = 2.792, * |
|  | **1-back** | | **30s *vs*.**  **60s** | contrast coefficient: 1.0900948  t value = 0.470, n. s. |
|  |  |  | **30s *vs*. 90s** | contrast coefficient: 9.4035654  t value = 4.013, *** |
|  |  |  | **60s *vs*. 90s** | contrast coefficient: 8.3134706  t value = 3.486, ** |
|  | **2-back** | | **30s *vs*. 60s** | contrast coefficient: 5.3842633  t value = 2.351, *. |
|  |  |  | **30s *vs*. 90s** | contrast coefficient: 13.0735514  t value = 5.579, *** |
|  |  |  | **60s *vs*. 90s** | contrast coefficient: 7.6892881  t value = 3.245, ** |
|  | **3-back** | | **30s *vs*. 60s** | contrast coefficient: 9.2837431  t value = 3.877, *** |
|  |  |  | **30s *vs*. 90s** | contrast coefficient: 21.3913577  t value = 8.985, *** |
|  |  |  | **60s *vs*. 90s** | contrast coefficient: 12.1076146  t value = 5.004, *** |
| **Interaction effect WM load * Duration** | | | | |
| **NO FEEDBACK** | **30 s *vs.* 60 s** | | **0-back *vs*. 1-back** | coefficient: -1.2366061  t value: -0.379, n. s. |
|  |  |  | **0-back *vs*. 2-back** | coefficient: -5.5307746  t value = -1.705 , n. s. |
|  |  |  | **0-back *vs*. 3-back** | coefficient: -9.4302544  t value = -2.842, ** |
|  |  |  | **1-back *vs*. 2-back** | coefficient: -4.2941685  t value = -1.318, n. s. |
|  |  |  | **1-back *vs*. 3-back** | coefficient: -8.1936483  t value = -2.458, * |
|  |  |  | **2-back *vs*. 3-back** | coefficient: -3.8994798  t value = -1.177, n. s. |
|  | **30 s *vs.* 90 s** | | **0-back *vs*. 1-back** | coefficient: -2.8530139  t value = -0.857, n. s. |
|  |  |  | **0-back *vs*. 2-back** | coefficient: -6.5229999  t value = -1.959, n. s. |
|  |  |  | **0-back *vs*. 3-back** | coefficient: -14.8408062  t value = -4.420, *** |
|  |  |  | **1-back *vs*. 2-back** | coefficient: -3.6699861  t value = -1.108, n. s. |
|  |  |  | **1-back *vs*. 3-back** | coefficient: -11.9877923  t value = -3.591, *** |
|  |  |  | **2-back *vs*. 3-back** | coefficient: -8.3178063  t value = -2.491, * |
|  | **60 s vs. 90 s** | | **0-back *vs*. 1-back** | coefficient: -1.6164077  t value = -0.478, n. s. |
|  |  |  | **0-back *vs*. 2-back** | coefficient: -0.9922253  t value = -0.294, n. s. |
|  |  |  | **0-back *vs*. 3-back** | coefficient: -5.4105518  t value = -1.589, n. s. |
|  |  |  | **1-back *vs*. 2-back** | coefficient: 0. 0.6241824  t value = 0.186, n. s. |
|  |  |  | **1-back *vs*. 3-back** | coefficient: -3.7941441  t value = -1.117, n. s. |
|  |  |  | **2-back *vs*. 3-back** | coefficient: -4.4183265  t value = -1.306, n. s. |
